# Breeding site fidelity is lower in polygamous shorebirds and male-biased in monogamous species

**DOI:** 10.1093/beheco/arac014

**Published:** 2022-04-01

**Authors:** Eunbi Kwon, Mihai Valcu, Margherita Cragnolini, Martin Bulla, Bruce Lyon, Bart Kempenaers

**Affiliations:** 1 Department of Behavioural Ecology & Evolutionary Genetics, Max Planck Institute for Ornithology, Eberhard-Gwinner-Str. 8, D-82319 Seewiesen, Germany; 2 Faculty of Environmental Sciences, Czech University of Life Sciences Prague, Kamýcká 129, 165 00 Prague, Czech Republic; 3 Department of Ecology and Evolutionary Biology, University of California, 130 McAllister Way, Santa Cruz, CA 95060, USA

**Keywords:** dispersal, mate fidelity, migration, return rate, sex-bias, site tenacity, shorebird, wader

## Abstract

Sex-bias in breeding dispersal is considered the norm in many taxa, and the magnitude and direction of such sex-bias is expected to correlate with the social mating system. We used local return rates in shorebirds as an index of breeding site fidelity, and hence as an estimate of the propensity for breeding dispersal, and tested whether variation in site fidelity and in sex-bias in site fidelity relates to the mating system. Among 111 populations of 49 species, annual return rates to a breeding site varied between 0% and 100%. After controlling for body size (linked to survival) and other confounding factors, monogamous species showed higher breeding site fidelity compared with polyandrous and polygynous species. Overall, there was a strong male bias in return rates, but the sex-bias in return rate was independent of the mating system and did not covary with the extent of sexual size dimorphism. Our results bolster earlier findings that the sex-biased dispersal is weakly linked to the mating system in birds. Instead, our results show that return rates are strongly correlated with the mating system in shorebirds regardless of sex. This suggests that breeding site fidelity may be linked to mate fidelity, which is only important in the monogamous, biparentally incubating species, or that the same drivers influence both the mating system and site fidelity. The strong connection between site fidelity and the mating system suggests that variation in site fidelity may have played a role in the coevolution of the mating system, parental care, and migration strategies.

## Teaser

Birds may return to the same breeding site to reunite with their old partner. For migratory bird species, whether to return to the same site as last year to breed or disperse to a new site depends on their mating system. Among 49 species of shorebirds from around the globe, species that form an exclusive breeding pair returned to the previous breeding site more than species where one sex mates with multiple partners.

## Introduction

In some species, individuals only disperse as maturing juveniles between their location of birth and their first breeding site (“natal dispersal”, Howard 1960), after which they show life-long fidelity to this first breeding location. In other species, however, dispersal can occur throughout life and each adult must decide whether or not to disperse to a new breeding site at the beginning of each breeding season (“breeding dispersal”, Greenwood and Harvey 1982). Individual decisions on whether to stay or disperse, and—if dispersing—on how far to move, determine properties at the population level, such as the probability of site fidelity and the dispersal propensity (Burnham 1993; Kendall and Nichols 2004).

Being faithful to a natal or previous breeding site can be beneficial if it 1) increases the likelihood of finding suitable breeding habitat and mates, 2) increases familiarity with local conditions, 3) increases the chance to mate with locally adapted individuals and hence reduces costs of genetic recombination, 4) increases the chance to remate with a former breeding partner (benefit of pair experience), and 5) avoids potential costs related to movement and to settlement in a new area (Hinde 1956; Waser and Jones 1983; Switzer 1993; Weatherhead and Forbes 1994; Hendry et al. 2003). On the other hand, dispersal might be the more favorable option, because it 1) can buffer against temporal variation and spatial asynchrony in habitat quality, 2) reduces the risk of inbreeding depression, and 3) reduces competition among kin (Howard 1960; Greenwood 1980; Greenwood and Harvey 1982; Clutton-Brock 1989; Dieckmann et al. 1999; Bowler and Benton 2005; Ronce 2007; Clobert et al. 2009; Duputié and Massol 2013).

Juveniles disperse from the site where they were born to their first breeding site and such natal dispersal typically covers larger distances than breeding dispersal (Greenwood and Harvey 1982; Paradis et al. 1998 and references therein). Thus, theoretical as well as comparative studies on the evolution of dispersal have focused more on natal dispersal (Perrin and Mazalov 1999; Sutherland et al. 2000; Mabry et al. 2013; Henry et al. 2016) or were inexplicit about the distinction (Travis et al. 1999; Bowler and Benton 2005; Ims and Andreassen 2005; Matthysen 2005). Although the distances moved can covary between natal and breeding dispersal (as shown in a study of 69 avian species; Paradis et al. 1998), the causes and consequences of the two are likely different and hence natal and breeding dispersal should be considered separately (Johst and Brandl 1999; Harts et al. 2016). Whilst the evolutionary drivers of natal dispersal (i.e., low natal philopatry) are well understood (avoiding inbreeding depression and kin competition), we still lack insight into the factors driving variation in breeding dispersal or, conversely, the extent of breeding site fidelity.

Breeding dispersal is often strongly sex-dependent (Greenwood 1980; Greenwood and Harvey 1982; Clarke et al. 1997). Multiple hypotheses suggest that this is because the two sexes differ in resource limitation, mating opportunities, competitive ability, morphological capacity to disperse, or even in the genetic basis of dispersal (Mabry et al., 2013; Trochet et al. 2016; Li and Kokko 2019). These sexual asymmetries vary with the social mating system and the intensity of sexual selection, and are linked to the level of sex bias in breeding dispersal (Greenwood 1980; Perrin and Mazalov 1999; Mabry et al. 2013; Brom et al. 2016). For example, in socially monogamous systems, males typically defend resources to gain mating opportunities and are less likely to disperse than females, whereas in polygynous and polyandrous systems, the sex being pursued (i.e., female and male, respectively) is less likely to disperse (Greenwood 1980). In general, the pattern of dispersal is opposite in birds and mammals, presumably due to the difference in the prevalent mating system: in birds, social monogamy, resource defence by males and female-biased dispersal are typical, whereas in mammals, social polygyny, female defence by males and male-biased dispersal are more common (Greenwood 1980).

Sex-biases in breeding dispersal and the underlying potential drivers have been examined across broad taxonomic groups, but the results have been less congruent in birds. For example, a review of 102 bird species from 31 families revealed that 40% of species show no sex-bias in breeding dispersal (while the rest showed female-biased dispersal, as expected; Clarke et al. 1997). Also, female-biased dispersal was linked to male territoriality in mammals, but not in birds (Trochet et al. 2016). Moreover, a recent comparative study on 86 bird species from 41 families showed that the sex-bias in breeding dispersal was not related to the social mating system, nor to other indices of the intensity of sexual selection (e.g., sexual size dimorphism, sex differences in parental care patterns, testis size; Végvári et al. 2018).

The potential reasons for these equivocal findings in birds are manifold. First, other factors that cause individual- or population-level variation in breeding dispersal may override the expected effects of sex at the population level, and previous studies often did not control for confounding factors. For example, dispersal behavior may be condition-dependent (Clobert et al. 2009; Ims and Hjermann 2001), may vary with population density (Travis et al. 1999; Kokko and Lundberg 2001; Matthysen 2005), or may have co-evolved with other traits under selection (Paradis et al. 1998; Saastamoinen et al. 2018). Second, breeding dispersal and sex bias in dispersal needs to be carefully defined. Many studies used the median or maximum dispersal distance reported for each species (e.g., Paradis et al. 1998; Sutherland et al. 2000; Serrano et al. 2021). However, one can also define it based on the proportion of individuals that dispersed. Importantly, dispersal distance does not necessarily covary with an individual's propensity to disperse (Hewison et al. 2021). Here, we use site fidelity to a breeding area as an indirect estimate of breeding dispersal propensity and explore variation in site fidelity within and among species of migratory shorebirds. We relate overall site fidelity and sex bias in site fidelity to two variables that reflect the intensity of sexual selection—the social mating system and sexual size dimorphism (Dale et al. 2015), while controlling for confounding factors such as the location of the breeding population.

Shorebirds have evolved one of the most diverse range of mating systems and the mating system is linked to sexual size dimorphism, parental care patterns, and migration strategies (Pitelka et al. 1974; Emlen and Oring 1977; Székely and Reynolds 1995; Borowik and McLennan 1999). Site fidelity varies widely among shorebirds: in some species, most individuals return to the same area, and some even breed in the same nest scrape from the previous year (Herzog et al. 2018), while in other species individuals rarely return to the same site to breed and move across continents even within a single breeding season (Kempenaers and Valcu 2017). An overall higher site fidelity has been reported for monogamous shorebird species regardless of sex (Oring and Lank 1984; *N* = 12 species), or for males regardless of the mating system (Tomkovich and Soloviev 1994; 5–11 species). Both studies drew attention to the apparent link between breeding site fidelity and sex-specific territoriality and parental roles, which vary with the mating system. However, a rigorous test of sex-bias in site fidelity and of the link between site fidelity and the intensity of sexual selection as reflected by the mating system and by sexual size dimorphism is lacking.

Based on previous studies (Oring and Lank 1984; Tomkovich and Soloviev 1994), we expect higher site fidelity for males than for females, and for socially monogamous species compared with non-monogamous species. We hypothesize that the sex-bias in site fidelity is linked to variation in the mating system, that is, to the sex-specific parental roles. Specifically, we predicted that 1) in socially monogamous species, males show higher site fidelity than females, because males defend their nesting territory, and 2) in socially polygamous species, the limiting sex (i.e., the incubating sex) shows higher site fidelity than the opposite sex (i.e., the one competing for mates), because the incubating sex will benefit more from having local experience, whereas the opposite sex will benefit more from freely relocating to find available mates.

## MATERIALS AND METHODS

### Estimating site fidelity

Site fidelity is defined as the probability that an individual returns to the same breeding site (local population) and does not permanently emigrate, if it survives (Sandercock 2003). True site fidelity of a population can only be estimated when we simultaneously estimate, and thus can disentangle the site fidelity from, the survival rate, breeding propensity, and detection probability (Souchay et al. 2014). Although sophisticated analytical methods are now available and have been used to estimate true site fidelity, such estimates are limited to a handful of species and populations (Sagar et al. 2002; Ledee et al. 2010; Cohen and Gratto-Trevor 2011; Catlin et al. 2015; Murphy et al. 2017; Hunt et al. 2018). Therefore, we used the most readily available proxy of site fidelity, the local return rate to a breeding population from one year to the next (hereafter, return rate).

We defined “return rate” as the proportion of adult birds marked in one year that was detected in the same study area in the next breeding season. Because the boundary of a study area or a breeding site is typically determined by the researcher, whether a bird returned or not becomes a scale-dependent issue (Barrowclough 1978). Here, we assume that the area of each study site was determined based on the distribution of the species of interest and represents a local breeding population. Nevertheless, we controlled for the effect of study area size in our analysis. Some of the observed variation in return rates is attributable to demographic parameters other than site fidelity, especially the annual survival rate (Payevsky 2016). Because survival rate is most strongly and consistently related to body size (Méndez et al. 2018; Weiser et al. 2018), we also controlled for body size. We only used return rates of adults (previous breeders), and assumed that the risk of not detecting an individual that had actually returned due to temporary emigration was low because most of the data come from comprehensive breeding monitoring studies.

### Data on return rates

**Figure 1:**
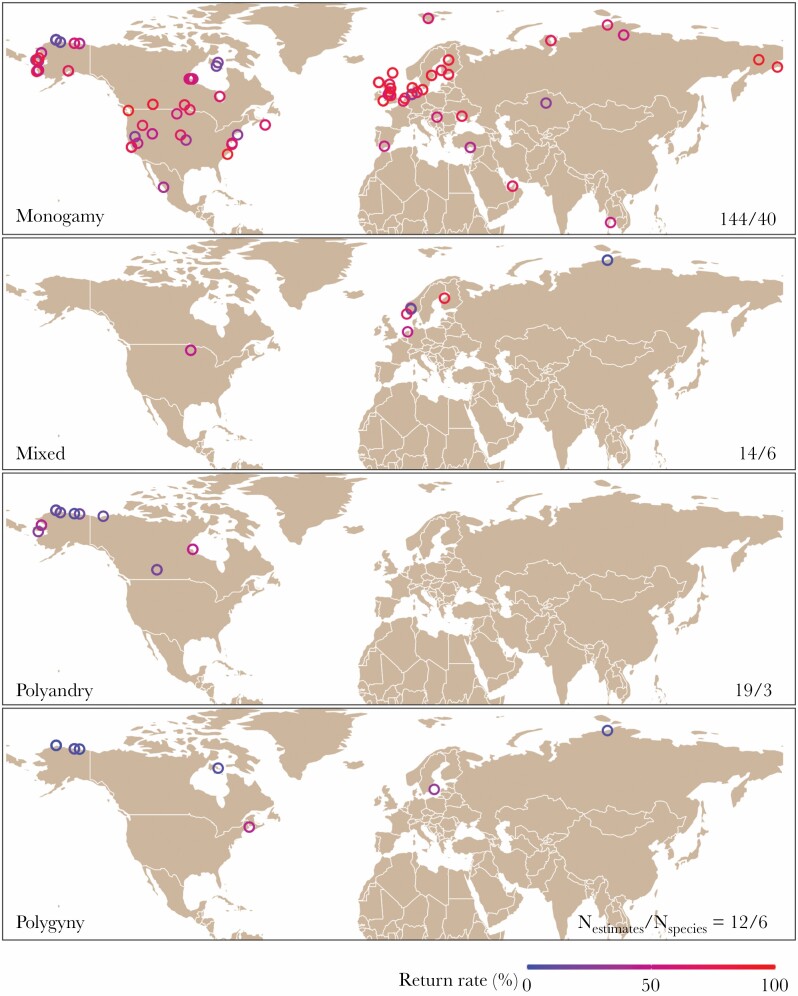
The geographical distribution of shorebird populations for which data on annual adult return rates were included in this study (see Methods for selection criteria). Each panel shows the data for a specific social mating system. “Mixed” denotes species in which both males and females have been reported to mate with more than one social partner within the same breeding season (whereby both partners can provide parental care) and is different from “monogamy” (with biparental incubation), “polyandry” (male-only care) and “polygyny” (female-only care).

Our study focuses on four families in the order Charadriiformes (Scolopacidae, contains 87 species, Charadriidae with 69, Recurvirostridae with 10, Haematopodidae with 11), which are conventionally categorized as “waders” or “shorebirds”. Return rates were extracted from 1) literature searches through Web of Science with the search phrase “SY = <SPECIES NAME> AND TS = (return OR fidel* OR philo*)”, 2) a general search on Google Scholar and backward citation searches, and 3) a direct estimation from the raw data (either collected ourselves or obtained with permission from others). We only considered return rates of adult birds to a breeding location north of the Equator, because the available data from the Southern Hemisphere was sparse. We only included return rate estimates from studies that marked more than 20 birds, and estimated return rates on a yearly basis. In other words, we excluded return rates that were estimated from all study years pooled (e.g., when reported as the percentage of the population that was resighted at least once during multiple study years). When a publication reported multiple estimates of year-specific return rates, we used the mean value to represent the return rate of the population. We included only individuals that did not carry any type of transmitter. We initially found 462 estimates of adult return rate to a breeding location from 74 species. However, the above-mentioned data filters led to a more restricted dataset (Figure 1; see Data analysis for the final sample size). For populations for which the return rate was reported separately for males and females, we calculated the sex bias in return rate as the return rate of males divided by the sum of return rates for the two sexes. Therefore, a value of 0.5 indicates no sex difference in return rates, a value greater than 0.5 indicates a male bias in return, and a value smaller than 0.5 indicates a female bias in return.

For each study, we extracted the following variables: the total number of birds marked (pooled across years), geographic coordinates of the breeding location, size of the study area, and number of study years during which birds were marked and resighted.

### Geographic predictors

Previous studies on shorebirds suggested that site fidelity is lower towards the edge of the breeding range (Ryabitsev and Alekseeva 1998; 12 species), at higher latitudes (Ryabitsev and Alekseeva 1998; Klima and Johnson 2005), or in species that migrate over larger distances (Klima and Johnson 2005; 31 species). We could not address the potential effects of migration distance on site fidelity due to the lack of information on population- and sex-specific migration routes for the species included here. However, to control for the other variables, we obtained the breeding range for each species (Valcu et al. 2012; BirdLife International 2021), and calculated three population-level predictors to include in the model: the breeding range span (in degrees), the relative latitude (in degrees) and the relative distance to the nearest breeding range boundary (in meters). Relative latitude indicates the position of a population in relation to the midpoint of the species’ latitudinal range span, with positive values indicating that the breeding population is further north than the centre of the range. Note that in our dataset this variable is correlated with the species’ latitudinal breeding range span (Pearson's *r* = −0.50), so we did not include range span in the model. The relative distance to the breeding range boundary indicates how close each breeding population is to its closest range boundary relative to other potential breeding locations. Further details and a visual description of the geographic predictors are provided in the Supplementary Material A.

### Life-history predictors

We categorized the social mating system of each species as monogamy, polyandry, polygyny, and mixed, based on the species’ mating strategy as well as parental behavior, described in the Birds of the World (https://birdsoftheworld.org/bow/home; Billerman et al. 2020), consultation of species experts, and additional literature. In brief, we considered a species socially monogamous if an exclusive pair bond exists and both members of a pair provide parental care, at least during incubation. Polyandry is defined as females mating with multiple males within a breeding season and not providing any form of parental care. Similarly, polygyny is defined as males mating with multiple females within a season and not providing parental care. We used the term “mixed” to denote systems with various degrees of polyandry and polygyny, but where both males and females can provide parental care (Oring 1986). Therefore, under our classification, serially polygamous species, such as the snowy plover *Charadrius nivosus* and the Kentish plover *C. alexandrinus*, were grouped under “monogamy” because both the male and the female typically incubate their first clutch together. Although a species may be classified as polyandrous or polygynous, the actual rate of polyandry and polygyny varies across years within a population, among populations within a species, or across the species’ geographic range (Pienkowski and Green 1976; Schamel and Tracy 1977; Oring et al. 1983). For example, not all female red phalaropes *Phalaropus fulicarius* are polyandrous, that is, achieve polyandry every breeding season. However, females never invest in parental care (Schamel and Tracy 1977, 1987). The same is true for polygamous species, but with female-only care (e.g., Lanctot et al. 1997). In contrast, in some populations or years all individuals in the “mixed” system might be socially monogamous (e.g., in the sanderling *Calidris alba*; Reneerkens et al. 2014). Because the mating system of the mixed group is highly variable and flexible at the individual level, we excluded this group from statistical analyses, but show the raw (descriptive) data in the figures for comparison. We obtained data on each species’ mean wing length (in mm: Dale et al. 2007; Klima and Jehl 2020; Pakanen, unpublished data), and calculated body size dimorphism as the difference between the log_10_-transformed average wing length of males and females.

### Data analyses

We constructed models to investigate variation in two response variables: return rate (*N* = 175 estimates from 111 populations of 49 species) and the sex bias in return rates (*N* = 65 estimates from 65 populations of 33 species). Both response variables vary between 0 and 1 and are beta distributed. Thus, we used a beta logistic regression model with a logit-link function in a Bayesian framework, and replaced values of 0 with 0.00001 and values of 1 with 0.99999 prior to analysis.

To model variation in return rate, we included sex, mating system, and log_10_-transformed wing length as fixed effects. We also included the potentially confounding variables relative latitude, relative distance to the range boundary, the year of study initiation, study duration (in years), the total number of marked birds (log_e_-transformed), and study area size (in ha, log_e_-transformed). Finally, we added species or subspecies as random effect. Subspecies were used for the Red knot (*Calidris canutus roselaari, C.c.canutus*), the Dunlin (*Calidris alpina schinzii, C.a.pacifica, C.a.arcticola*), and the Willet (*Tringa semipalmata semipalmata, T.s.inornata*), as these may have distinctive migration strategies.

To model variation in sex differences in return rate, we included mating system and sexual size dimorphism (i.e., the difference between the log_10_-transformed average wing length of males and females) as fixed effects, and (sub)species as random effect. We did not control for other factors, because the sexes are compared within the same population, and we assumed that geographic factors and study-specific conditions affected males and females similarly. Because sexual dimorphism strongly correlates with the social mating system (Dunn et al. 2001), we ran two additional models that included either the mating system or sexual size dimorphism as a single fixed effect and compared the results with those of the full model. Furthermore, we weighed each observation by the scaled sample size (i.e., the total number of birds banded for each population).

Prior to modeling, all continuous explanatory variables were standardized by subtracting the mean and dividing by the standard deviation. We examined potential multicollinearity among explanatory variables in two ways. 1) We checked the correlation matrix, which suggested that correlations between variables were relatively small (Fig. S1). 2) We calculated a variance inflation factor (VIF) for each predictor, whereby VIF values > 2 indicate multicollinearity (Zuur et al. 2010). All predictors in our models had a VIF < 1.5, indicating limited multicollinearity.

Various statistical methods can be used to control for phylogeny in comparative analyses (Rohlf 2001), but it has been argued that the interpretation of these methods has not always been correct (Rohle 2006). Recently, Uyeda et al. (2018) pointed out that the need for phylogenetic control depends on the phylogenetic signal in the residuals of the model and not in the response variable. The authors stated that “…if all of the phylogenetic signal in a data set is present in the predictor trait and the errors are independent and identically distributed, then there is no need for any phylogenetic correction” (Uyeda et al. 2018). After recent recommendations (Hansen 2014; Uyeda et al. 2018), we tested whether the residual variance showed a significant phylogenetic covariance. For each model, we used the residual error from the full model as our new response variable and fitted a Bayesian linear regression model with an intercept only and with species and their phylogenetic relatedness as random effects (see Supplementary Material B for details) to reveal whether phylogeny explains any left-over variation in the data. Additionally, we ran a Bayesian hypothesis test comparing the above-described model against the same model without the random effect of phylogeny (Wagenmakers et al. 2010). We compared the model with and without phylogeny using the Bayes factor, which indicates strong evidence favoring the alternative hypothesis when > 10 (Lee and Wagenmakers 2014). We found that 1) phylogeny explained little variation in the residuals for both models (for return rate: 0.08, 95% CI: 0.00–0.24; for sex difference in return rates: 0.13, 0.01–0.40), and 2) the Bayes factor equaled 31.1 (return rate) and 17.4 (sex difference in return rates), respectively, in favor of a non-phylogenetic model (see Supplementary Material B for details). Thus, we did not use a phylogenetic comparative method for the main analyses.

We ran the Bayesian models with the probabilistic programming language STAN (Stan Development Team 2020) through the R package “brms” v. 2.14.4 (Bürkner 2017) in R v. 4.0.2 (R Core Team 2020). We sampled from 5 chains of 50 000 iterations each and used the first 25 000 iterations as burn-in. We then saved the output from every fifth iteration to avoid autocorrelation, resulting in 25 000 estimates to generate posterior distributions of parameters. We increased the target average proposal acceptance probability from 0.95 to 0.99 so that our sampling is more conservative to posterior distributions with high curvature (Bürkner 2017). We generated priors using the “get_prior” function in “brms”, which sets non-informative priors for all slope coefficients and uses a Student’s *t* distribution for the intercept and standard deviation, and a gamma distribution for phi (Bürkner 2017). For each model, we checked the convergence by visually checking the trace plot (Fig. S2 & S3) and using the Gelman-Rubin convergence diagnostics, which was < 1.1 for all estimates of model parameters (values approaching 1 indicate that the estimated between- and within-chain variances for model parameters are similar and hence indicate model convergence; Brooks and Gelman 1998).

## RESULTS

**Figure 2:**
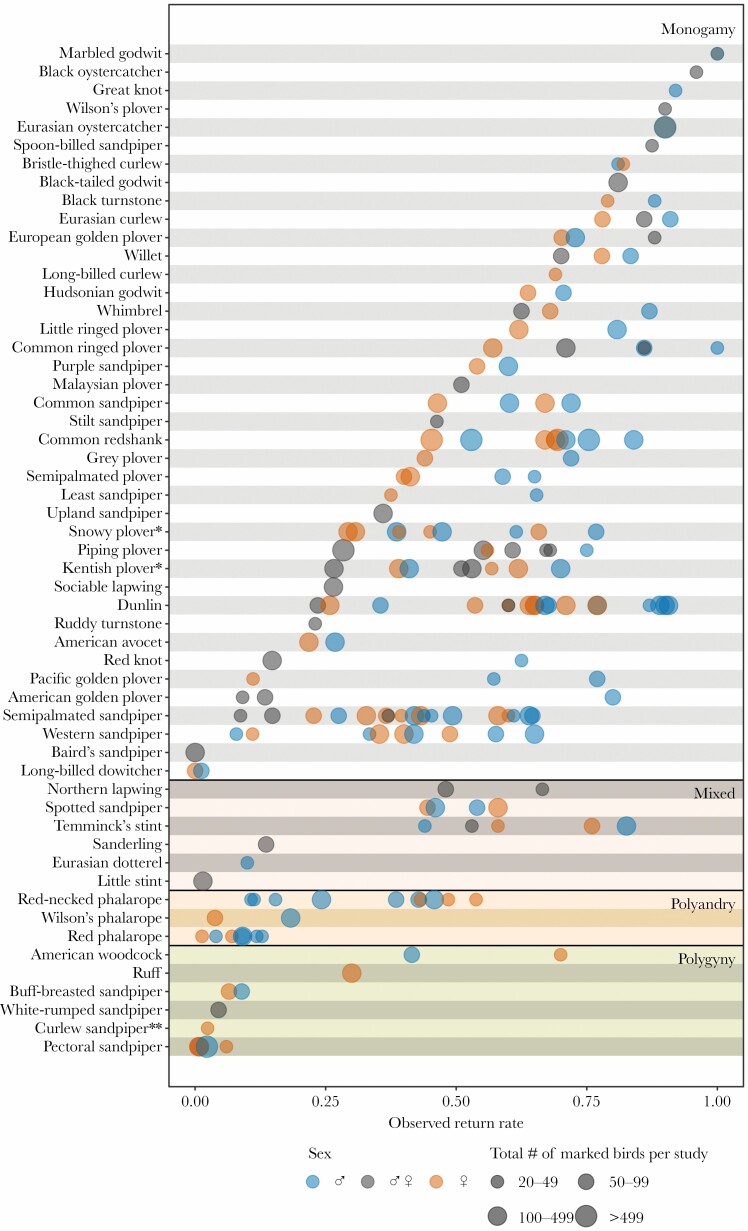
Observed return rates for 55 species of shorebirds with different social mating system. Each dot indicates an observed return rate for a given population (*N* = 120 populations), whereby dot size indicates the total number of marked individuals in the study, and color refers to sex (gray = sex unknown or sexes combined). Species are listed in descending order of average return rates within each mating system category. *Snowy plovers and Kentish plovers are typically classified as serially polygamous, but considered monogamous in this study because they normally maintain a pair bond for the first clutch and both pair members incubate the eggs. **The mating system of the curlew sandpiper is unknown, but suspected to be polygyny from observations of female-only incubation and early departure of males from the breeding grounds (Holmes and Pitelka 1964).

**Figure 3:**
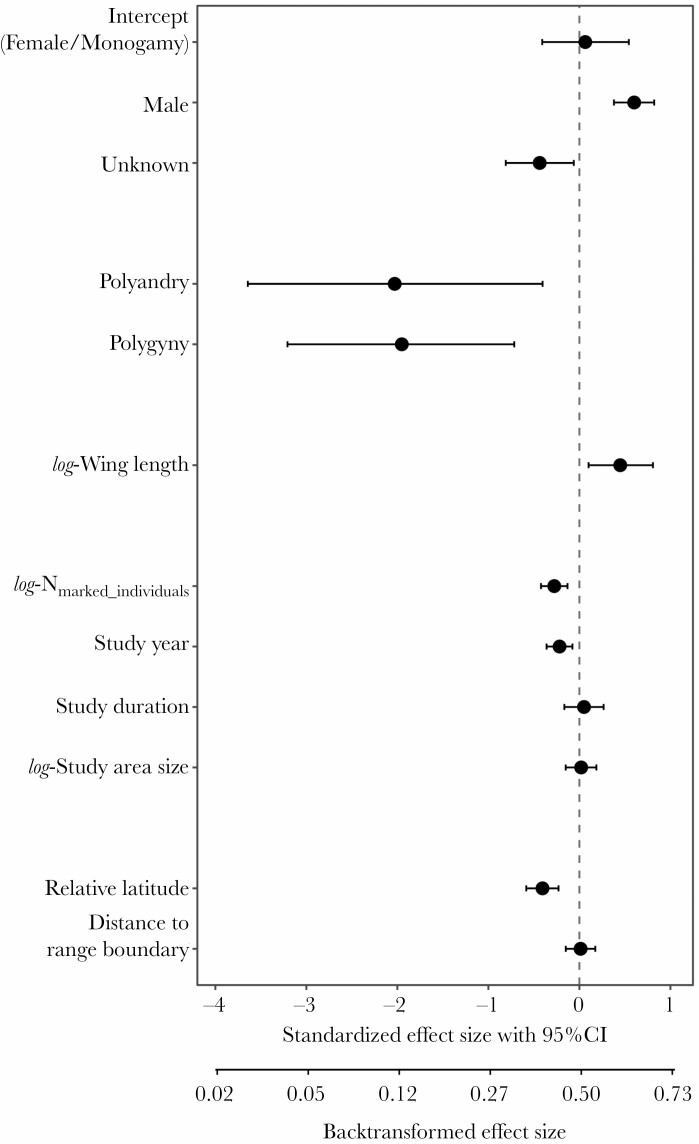
Standardized effect sizes (posterior means) of predictors explaining variation in annual return rates of 49 species of shorebirds (*N* = 175 estimates from 111 populations; sample sizes are different from Figure 1 because the “Mixed” group is excluded from the analysis). Error bars indicate 95% Bayesian credible intervals. A predictor has a significant effect if the 95% CI does not overlap zero. “Female” and “social monogamy” are the reference group.

Return rates varied widely among species, from zero (female long-billed dowitcher *Limnodromus scolopaceus*, *N* = 63 marked individuals, Utqiaġvik) to 1 (male and female marbled godwit *Limosa fedoa*, *N* = 57 individuals, South Alberta; Figure 2). Mating system was the strongest predictor of variation in return rate: on average 51.5% of all individuals returned in monogamous species, compared with only 11.7% in polyandrous species and 12.5% in polygynous species (Figures 2 and 3, Table S1). In general, return rates were higher for males (on average 64.6%) than for females (51.5%; Figures 2 and 3). As expected, return rates were higher for larger species, but the effect size was relatively small (Figure 3, Table S1). Return rates were lower for populations further north within the breeding range (Figure 3, Table S1). A sensitivity analysis showed that the estimated effect sizes were robust to varying cutoffs for the minimum number of individuals in a population (Fig. S4).

**Figure 4:**
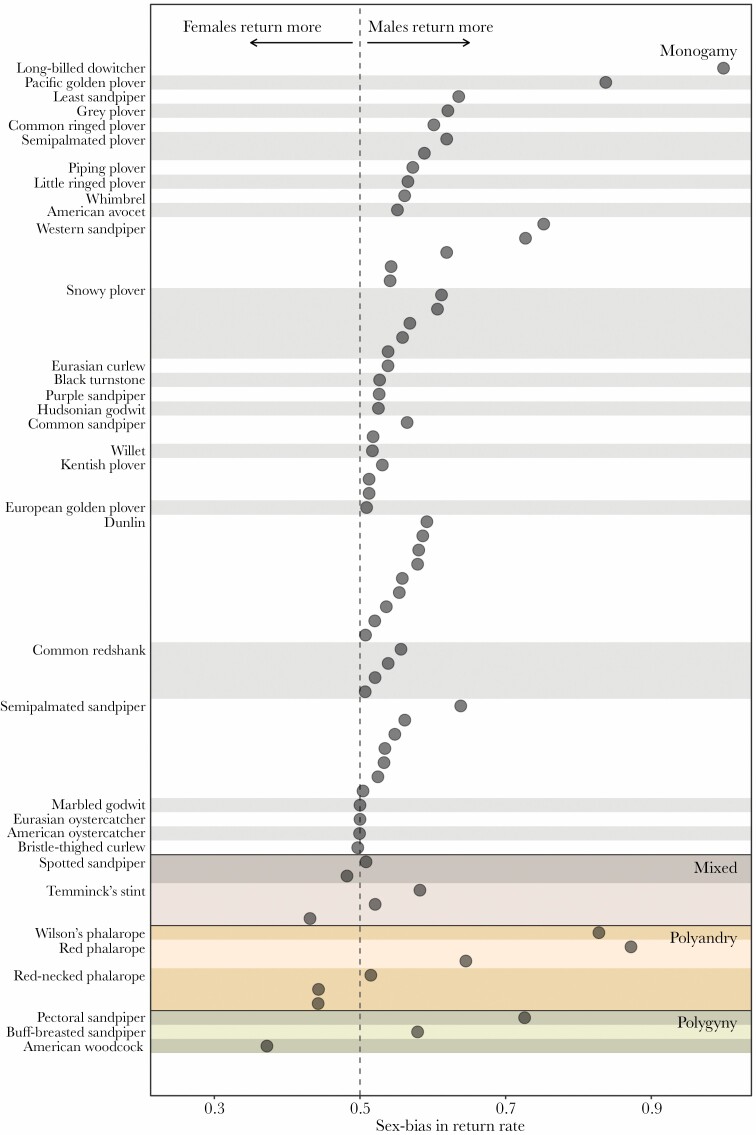
Observed sex bias in annual return rate of 35 species of shorebirds with different social mating system. Estimates for each population are shown separately (*N*_total_ = 70 populations). Species are listed in descending order of observed mean sex bias in return rates within each mating system category.

**Figure 5:**
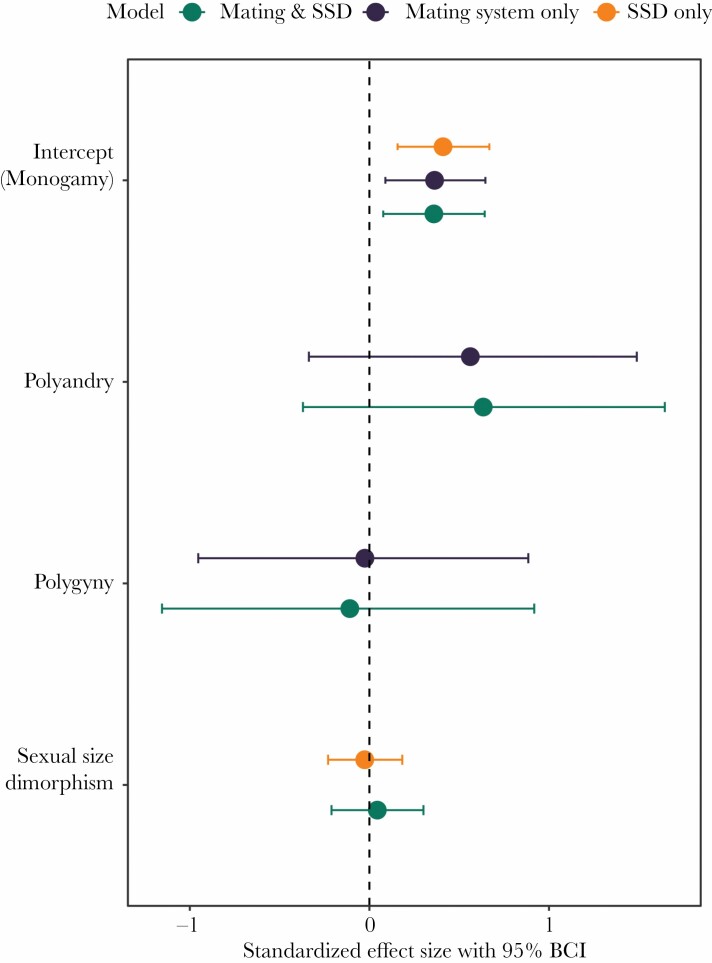
Standardized effect sizes (posterior means) of predictors on the sex bias in annual return rates of 33 species of shorebirds (*N* = 65 estimates from 65 populations; sample sizes are different from Figure 4 because the “Mixed” group is excluded from the analysis). We assessed the effect of the mating system and of sexual size dimorphism (SSD) by adding them as fixed effects in combination or separately (three models, as indicated by color). Error bars indicate 95% Bayesian credible intervals. A predictor has a significant effect when the 95% CI does not overlap zero. “Social monogamy” is the reference group.

Sex differences in return rate within each population also varied considerably, from a 28.5% higher return rate in females (American woodcock *Scolopax minor*) to a 46.1% higher return rate in males (Pacific golden plover *Pluvialis fulva*; Figure 4). Sexual dimorphism in wing length was strongly correlated with the mating system (one-way ANOVA: F_2,61_ = 11.14, *P* < 0.001), such that females were larger than males in polyandrous species and vice versa in polygynous species. However, neither mating system nor sexual dimorphism in wing length predicted sex differences in return rate (Figure 5, Table S2). The lack of a relationship between mating system and the sex-bias in return rates remained when we treated the latter as a binary response variable (i.e., 0 if females return more than males and 1 if males return more than females; Table S2).

## Discussion

Our data show that return rates and the sex bias in return rates vary widely between shorebird species and populations. Part of this variation might be due to differences in survival, as indicated by a significant positive effect of body size (our study, also see Méndez et al. 2018; Weiser et al. 2018). However, even after controlling for this effect, return rates of both sexes depended on the social mating system and, as predicted, were strongly male-biased in monogamous species.

Based on data from 111 populations of 49 shorebird species, return rates were significantly lower for polyandrous and polygynous species compared with monogamous species (Figure 3). Hypotheses on the evolution of sex-biased dispersal can explain why the competing sex of polygamous mating systems would benefit from dispersing more, but they do not explain why the return rates of the limiting sex (i.e., males of polyandrous and females of polygynous species) are lower than those of monogamous birds. The low return rate of the limiting sex (i.e., incubating sex) in polygamous systems can be explained by three evolutionary scenarios. 1) The benefits of dispersal may be higher for both sexes. In polygamous shorebirds, the number of breeders at a given site often fluctuates greatly between years (Pitelka et al. 1974; Troy 1996). This may have arisen because the incubating sex benefits from dispersal, for example, because of yearly variation in local habitat quality. Heterogeneity in the spatiotemporal distribution of the dispersing sex could then have increased the benefit of dispersal for the opposite sex as well in pursuit of finding a mate. This could have led to a positive feedback loop leading to increased dispersal propensity for both sexes. 2) The benefit of being site faithful might be lower in polygamous species. This seems counterintuitive, because in uniparental species, a direct benefit of breeding in a familiar environment with local knowledge, for example, about suitable foraging areas should be higher for the caring sex (Widemo 1997). On the other hand, a key benefit of returning to the same site, namely the opportunity to reunite with a social mate from the previous year, is irrelevant for uniparental species (see below). 3) The high dispersal tendency of one sex may constrain the evolution of high site fidelity in the other sex. Given that dispersal propensity presumably has a genetic basis (Doligez et al. 2009; Saastamoinen et al. 2018), the low return rates of one sex may be a by-product of strong selection favoring dispersal in the competing sex. However, this is true independent of the mating system, and would lead to no or reduced sex bias in dispersal.

To understand the evolution of low site fidelity in polygamous species, we can also consider the selection pressures favoring high site fidelity in monogamous species and examine whether those selection pressures are missing in polygamous species. Several studies on monogamous shorebirds suggest a strong correlation between site fidelity and reproductive success based on observations that 1) successful breeders are more likely to return (long-billed curlew *Numenius americanus*; Redmond & Jenni 1982, common redshank *Tringa totanus*; Thompson & Hale 1989, black turnstone *Arenaria melanocephala*; Handel & Gill 2000), and that 2) males with prior site experience have higher fitness (western sandpiper *Calidris mauri*; Johnson & Walters 2008). However, evidence for a causal relationship between site fidelity and reproductive success is largely missing, and requires showing decreased reproductive success of dispersers at the new breeding sites (piping plover *Charadrius melodus*; Saunders et al. 2012). Fitness benefits of high site fidelity may arise from accumulated experience with the local environment or with the mate, or it may simply reflect an age-related improvement in performance. Two studies that aimed to discern between these effects found that the fitness benefit of prior site experience arose from breeding earlier in the season (Johnson and Walters 2008) or from taking a shorter time to renest after failure (van Leeuwen and Jamieson 2018). In both studies, the advantage of breeding site fidelity came from the time saved by mating with the previous partner rather than from the site experience per se. This so-called “fast-track hypothesis” may also be the driver of long-term monogamy in other systems (e.g., in wild zebra finches *Taeniopygia guttata*; Adkins-Regan & Tomaszycki 2007).

The fast-track hypothesis states that the benefit of returning to the same site lies in mate reunion allowing early breeding, and predicts low site fidelity for both sexes of polygamous species, because 1) no or only short-term pair bonds are formed in these species and 2) selection favoring high dispersal of one sex implies that there will no longer be a reason to return to the same site for the opposite sex. It is interesting to note that polygamous species generally breed later in the season than monogamous species at the same site (Whitfield and Tomkovich 1996; Saalfeld and Lanctot 2017). One hypothesis to explain this observation is that polygamous species are “forced” to breed later, because polygamous males provide less or no care to females (Yom-Tov 1992). However, this hypothesis is based on observations of passerine species in which monogamous males provide food to their mate, which does not apply to shorebirds. For the incubating sex of polygamous shorebirds, the energetic need for self-maintenance directly trades off against incubation. Therefore, a more likely hypothesis is that uniparental incubation may be harder under harsher environmental conditions earlier in the season, especially in the Arctic where many shorebirds breed. Yet another alternative hypothesis is that polygamous species are not as constrained by the timing of breeding (for reasons that remain unknown), and hence, individuals do not need to “race” back to the previous breeding site. Although these hypotheses still need empirical testing, the general pattern of delayed breeding in polygamous shorebirds suggests that the lack of site fidelity might be linked to relaxed selection on the initiation of breeding in these species.

If the fast-track hypothesis is true and high site fidelity evolved as a means to reunite with the previous breeding partner, we would expect a strong connection between mate and site fidelity. Cézilly et al. (2000) reported a significant relationship between site fidelity and divorce rate in 42 species of Ciconiiforms (11 of which were also included in our study). Cézilly et al. (2000) suggested that the likely evolutionary pathway was first gaining site fidelity and subsequently mate fidelity. Note that in their study birds were considered “site faithful” only when returning to within 11 meters from the previous nest. Their results favor the idea that mate fidelity initially evolved as a by-product of site fidelity (rather than site fidelity being the strategy to maintain pair bonds). However, as the authors underscored in their paper, this transition only corresponds to the initial evolution of site fidelity, and they suggest that site and mate fidelity have been frequently lost and regained in recent times (Cézilly et al. 2000). The large variation in return rates seen in our data also suggests that migration or breeding site sampling processes can shift rapidly and reversibly over evolutionary time (Zink 2011). For example, the ruff *Calidris pugnax*, the pectoral sandpiper *Calidris melanotos*, and the great knot *Calidris tenuirostris* are closely related species with similar body size, and breed at similar latitudes. However, male return rates are vastly different, with 92% of great knots (*N* = 26) returning versus 33% of ruffs (*N* = 12) and only 1.5% of pectoral sandpipers (*N* = 891) (Scheufler and Stiefel 1985; Tomkovich 1996; Kempenaers, unpublished data). The most obvious difference between the three species is that the great knot breeds monogamously, whereas the ruff and pectoral sandpiper are polygynous with female-only care.

The early observations of Greenwood (1980), and both theoretical and empirical studies that followed (Mabry et al. 2013; Trochet et al. 2016; Li and Kokko 2019), predict male-biased breeding site fidelity under monogamy and polyandry and female-biased breeding site fidelity under social polygyny. The idea behind this is that the sex that defends resources would show higher site fidelity. Our results only partly support these predictions (Figure 5). As predicted, return rates were strongly male-biased in monogamous species. All 56 populations of 27 monogamous species showed a male-biased return (i.e., female-biased dispersal), except for one case of a weak female-bias in return (in the bristle-thighed curlew *Numenius tahitiensis*, 81% of the males returned and 82% of the females, Marks et al. 2020; Figure 4). These findings support the original idea that monogamous males that acquire mates by defending territories show higher fidelity to a breeding location. However, we found no support for a male-biased return rate in polyandrous species or a female-bias in polygynous species. Although the data are limited, the sex-bias in return rates in the non-monogamous systems was highly variable (Figures 4 and 5). The fact that non-monogamous species generally have low return rates suggests that benefits related to local experience or former residency are not the only reason why males and females of monogamous species return. The main difference is that individuals from polygamous species would not benefit from reuniting with a previous partner.

An important assumption of our study is that the survival rate is independent of the mating system, and therefore that variation in return rates and in sex bias in return rates reflect dispersal rather than survival. A recent review using mark-recapture and dead-recovery data showed significantly lower survival rates for females across 37 shorebird species (Mendez et al. 2018), despite the general trend that females are larger than males. This result fits the general idea that females have higher mortality because they are the heterogametic sex (ZW) in birds (Maklakov and Lummaa 2013). However, most survival estimates are confounded with female-biased dispersal. In fact, after accounting for sex-specific dispersal distances, the estimated true survival rates did not differ between the sexes in American golden plover *Pluvialis dominica*, dunlin *Calidris alpina*, semipalmated sandpiper *Calidris pusilla*, red phalarope *Phalaropus fulicarius*, and red-necked phalarope *Phalaropus lobatus* (Weiser et al. 2018). Similarly, no sex difference in survival was found in other groups in which females are larger than males, such as raptors (Newton et al. 2016). Therefore, we interpret the sex bias in return rates shown in our study as a true sex bias in site fidelity rather than differential survival. However, the observed return rates may still be lower than the true site fidelity (Martin et al. 1995), because of variation in the probability of detection, which depends on the sex and sex-specific behaviors (Sandercock 2003 and references therein). However, we note that none of the four confounding variables related to data quality (observation intensity) showed a strong effect on return rate (see Figure 3). Moreover, in polygamous shorebirds, individuals of the caring sex are often found through intense nest searching, while individuals of the competing sex are often highly detectable because of their conspicuous courtship behavior (Lanctot et al. 1998; Lesku et al. 2012), which might reduce a sex bias in detection.

This study shows the full spectrum of variation in return rates to previous breeding locations among 49 species of shorebirds. Local return rates varied by sex, with body size, and with relative breeding latitude, but it is most strongly related to the mating system. Our study indicates a strong connection between species-specific site fidelity and the social mating system, with monogamous species generally having higher return rates. The available evidence suggests that the benefit of breeding site fidelity might be linked to mate fidelity, allowing earlier breeding in a given season. Variation in the degree of site fidelity may have played a role in the coevolution of the mating system, the pattern of parental care, and migration strategies in shorebirds.

## FUNDING

Research support was provided by the Alexander von Humboldt Foundation (to E.K.) and the Max Planck Society (to B.K.).

We are grateful to all shorebird biologists who reported return rates in the 126 publications featured in our study. We especially thank Brendan Norman, Cheri Gratto-Trevor, Christian Hoefs, Dan Sullins, Emily Weiser, Jim Johnson, John Atle Kålås, Luke Eberhart-Hertel, Nathan Senner, Richard Lanctot, Rose Swift, Tiago M.G. Rodrigues, Veli-Matti Pakanen, and Willow English for sharing their data and knowledge on certain species. We thank Luke Eberhart-Hertel, Richard Lanctot and an anonymous reviewer for constructive comments on the manuscript.

## Supplementary Material

arac014_suppl_Supplementary_Material

## Data Availability

Analyses reported in this article can be reproduced using the data provided by Kwon et al. (2022).

## Data sources for annual return rates

Any source for a particular data point can be indexed within the supplementary data (Kwon et al. 2022).
