# Supplementary material for: Breeding site fidelity is lower in polygamous shorebirds and male-biased in monogamous species
Source: Behav Ecol. 2022 Apr 1;33(3):592–605. doi: 10.1093/beheco/arac014 (PMC9113309; doi:10.1093/beheco/arac014)
Supplement: arac014_suppl_Supplementary_Material [file arac014_suppl_supplementary_material.docx]

**Supplementary material**

**A.** **Estimation of the three geographic indices**

For every species for which we obtained an estimate of return rate, we determined their breeding range based on the BirdLife International Database (Valcu et al. 2012, BirdLife International 2017). To illustrate our methods, we use the breeding range of the common sandpiper as an example.


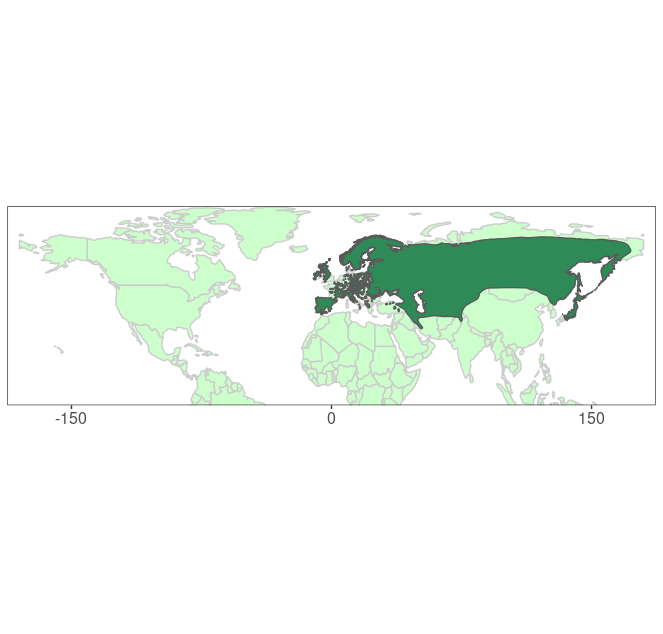


Based on the geospatial traits of each species’ breeding range and the location of the study population from which the return rate data came, we calculated three geographic indices: relative distance to the nearest range boundary, latitudinal range span, and relative latitude.

***Relative distance to the nearest range boundary*** is a measure that shows how close the study population is to the edge of the species’ breeding range. To estimate it, we first extracted the range border using the ‘st_cast’ function in the R package ‘sf’ (Pebesma 2018).

border = sf::st_cast(range, "MULTILINESTRING")


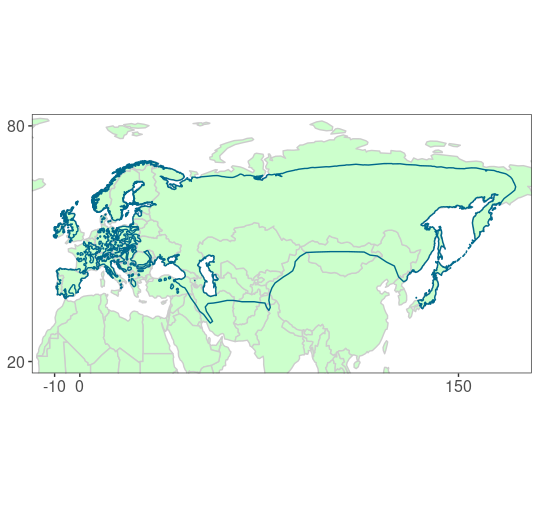


We then created a grid of 10000 points that covers the entire breeding range.

grid = st_sample(range, 10000, type = 'regular', exact = TRUE)


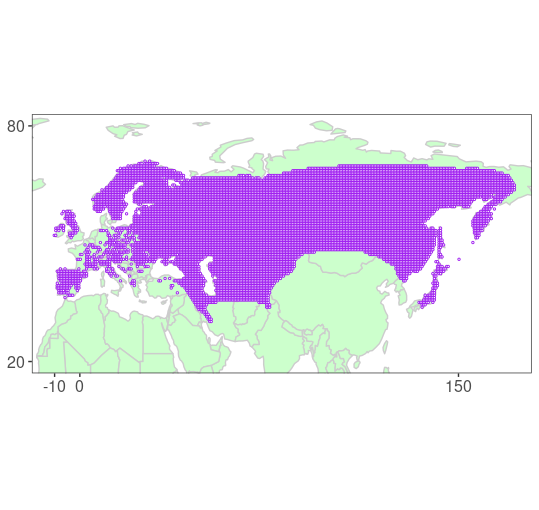


Then, we calculated the distance (in meters) from each point of the grid to its closest boundary.

dist = st_distance(border, grid)


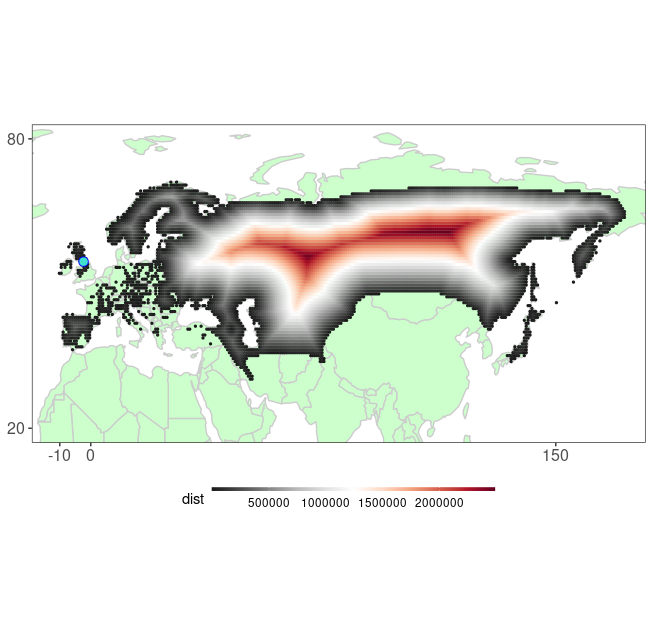


The above figure shows the breeding range map of the common sandpiper colored by distance from the nearest range boundary, with dark red indicating the area furthest away from a boundary, and the blue dot indicating the study population.


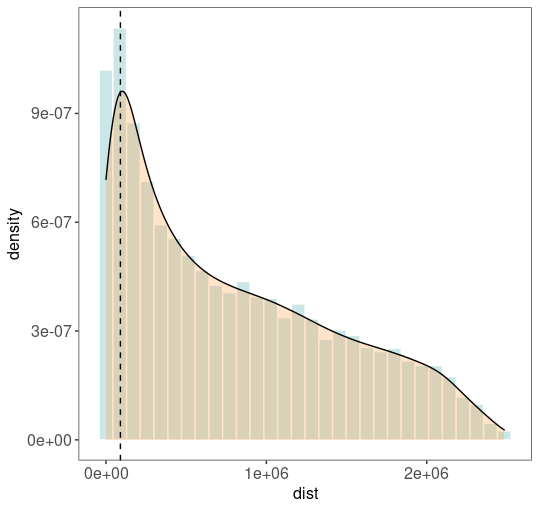


The figure above shows the distribution of nearest range boundary distances from the 10000 sampled points. Given the shape of the breeding range, there are more points close to a range boundary (left side of the distribution) than points that are close to the center (right tail of the distribution).

One of the return rate estimates was from a breeding population of common sandpipers in the UK (blue dot on the map above). We extracted the distance measure of one of the 10000 sampled points, namely the one closest to the latitudinal location of the breeding population. We then calculated the relative distance to the nearest range boundary of the focal population as the quantile to which the extracted distance belongs in the above data distribution (here: the 0.071th quantile).

Thus, our measure of distance to the nearest range boundary takes into account variation in the shape of the breeding range between species.

***Latitudinal range span*** was calculated as the straight-line distance (in meters) between the latitude of the most northern and the most southern point of the breeding range of a particular species.


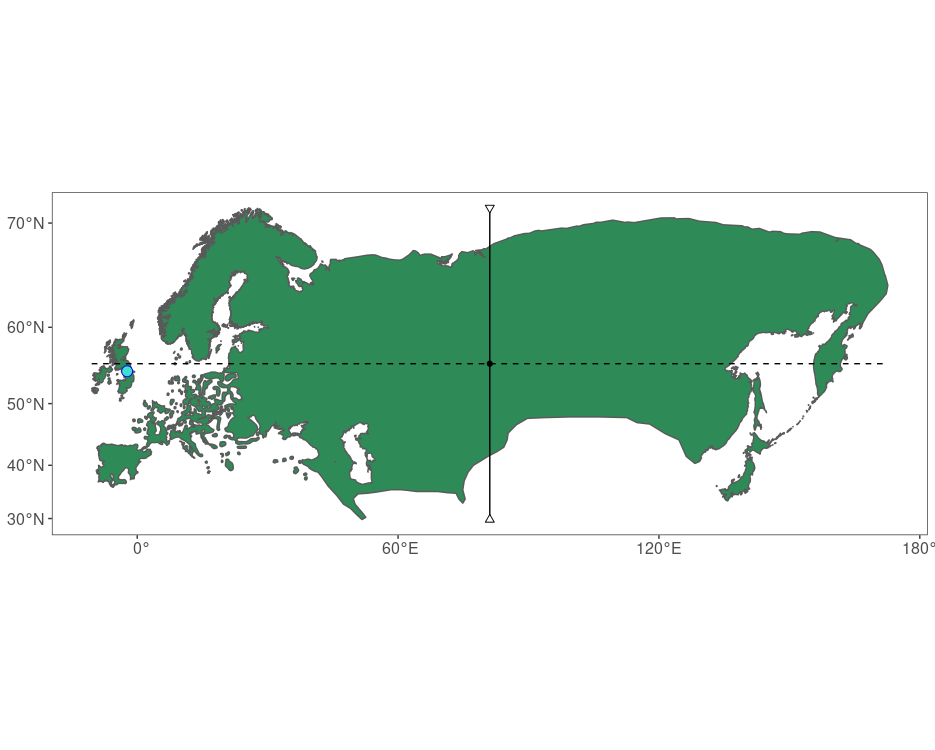


Latitudinal center line

Latitudinal span


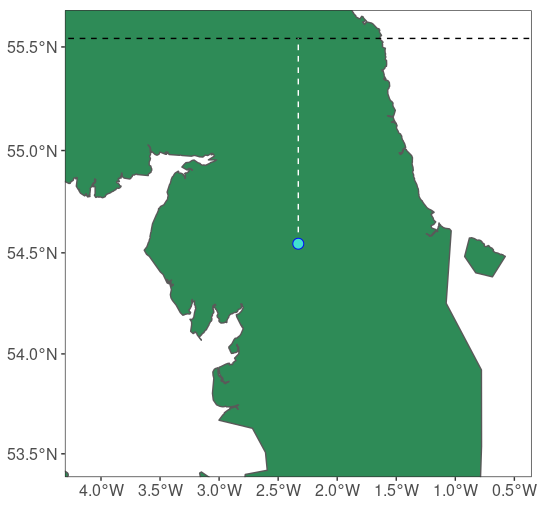


Relative latitude

***Relative latitude*** was calculated as the latitude of the study population relative to the latitude of the center of the breeding range. Thus, negative values indicate southerly populations within a species’ breeding range.

**References**

- BirdLife International. (2017). BirdLife International and handbook of the birds of the world. Bird species distribution maps of the world.
- Pebesma, E. (2018). Simple features for R: Standardized support for spatial vector data. *The R Journal*, 10, 439–446. https://doi.org/10.32614/RJ-2018-009
- Valcu, M., Dale, J., & Kempenaers, B. (2012). rangeMapper: A platform for the study of macroecology of life-history traits. *Global Ecology and Biogeography*, 21, 945–951.

**B.** **Phylogenetic signal on return rates**


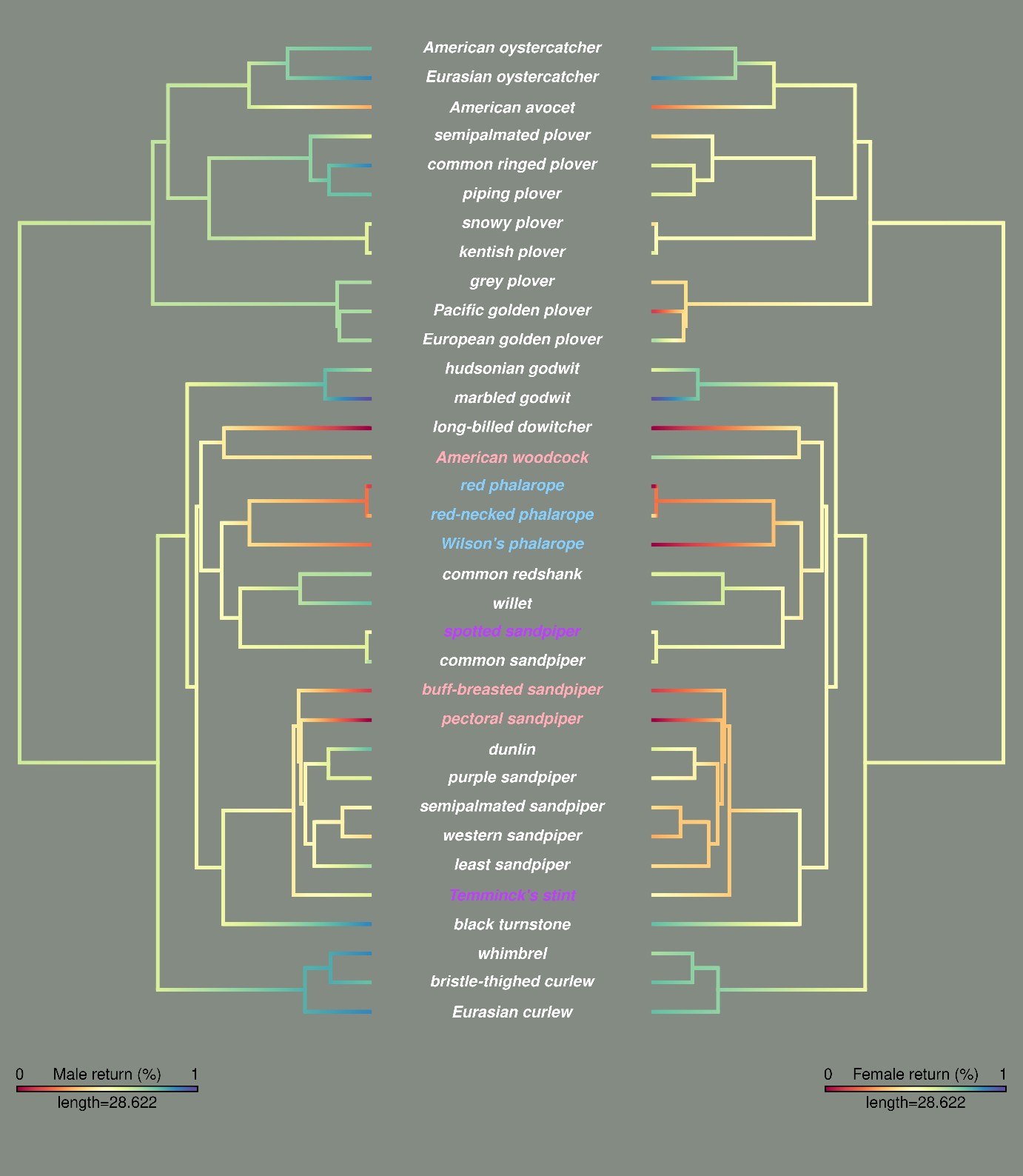

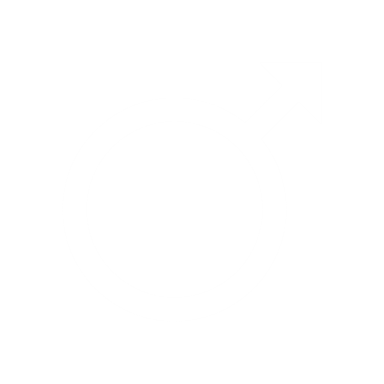

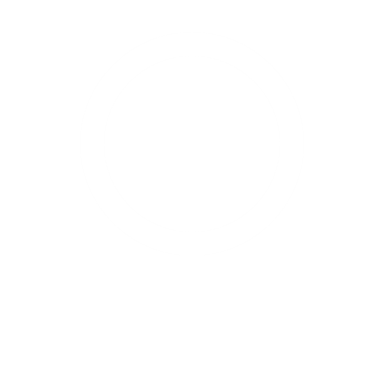


Mating system –– *Monogamy*

*Mixed*

*Polygyny*

*Polyandry*

**Figure**. Ancestral character estimation of annual return rate to a breeding site plotted on the phylogenetic tree for 34 species of shorebirds in the order Charadriiformes. Species mean return rates are shown separately for males (left) and females (right) and are illustrated by colors ranging from red (low return rate) to blue (high return rate). Phylogeny data are from Birdtree.org (Jetz et al., 2012) and the plot was created using the R package ‘phytools’ (Revell 2012). “Length” in the legend gives a scale for the branch lengths of the tree, in units of substitution per site following Jetz et al. (2012).

**Methods**

We sampled 100 phylogeny trees with Hackett backbone from Birdtree.org (Jetz et al. 2012) for 175 species that belong to four families in the order Charadriiformes (Scolopacidae, Charadriidae, Recurvirostridae, Haematopodidae). On each of the 100 trees, we manually added a branch for each of the four species that were missing, based on our best knowledge of the species’ phylogeny. Thus, we added a branch for spotted sandpiper (*Actitis macularia*) next to the common sandpiper (*Actitis hypoleucos*), snowy plover (*Charadrius nivosus*) next to Kentish plover (*Charadrius alexandrinus*), stilt sandpiper (*Micropalma himantopus*) next to curlew sandpiper (*Calidris ferruginea*), and red phalarope (*Phalaropus fulicaria*) next to red-necked phalarope (*Phalaropus lobatus*), using the bind.tree function in ape 5.4.1 (Paradis & Schliep 2019). We generated a consensus phylogeny from the revised 100 trees using the maxCladeCred function in phangorn 2.5.5 (Schliep 2011). The consensus tree was then used to test for a phylogenetic signal and to determine whether we needed to incorporate phylogeny in our model.

**Results**

The summary below shows the results of an intercept model with phylogeny fitted to the residual variance in return rates. Group-level effects for ‘phylogeny’ correspond to the random effect of phylogeny. The estimate of the standard deviation of the random effect of phylogeny was close to zero (0.08) and the 95% CI included zero, indicating that the residuals were not substantially confounded by phylogeny.

> summary(fm4.res.wP)

Family: gaussian

Links: mu = identity; sigma = identity

Formula: Estimate ~ 0 + Intercept + (1 | species) + (1 | gr(phylogeny, cov = A))

Data: x (Number of observations: 173)

Samples: 5 chains, each with iter = 5000; warmup = 2500; thin = 1;

total post-warmup samples = 12500

Group-Level Effects:

~species (Number of levels: 48)

Estimate Est.Error l-95% CI u-95% CI Rhat Bulk_ESS Tail_ESS

sd(Intercept) 0.06 0.05 0.00 0.17 1.00 8307 4789

~phylogeny (Number of levels: 48)

Estimate Est.Error l-95% CI u-95% CI Rhat Bulk_ESS Tail_ESS

sd(Intercept) 0.08 0.06 0.00 0.24 1.00 6857 5702

Population-Level Effects:

Estimate Est.Error l-95% CI u-95% CI Rhat Bulk_ESS Tail_ESS

Intercept -0.02 0.08 -0.18 0.14 1.00 8725 6680

Family Specific Parameters:

Estimate Est.Error l-95% CI u-95% CI Rhat Bulk_ESS Tail_ESS

sigma 0.76 0.04 0.68 0.85 1.00 16715 9073

Samples were drawn using sampling(NUTS). For each parameter, Bulk_ESS

and Tail_ESS are effective sample size measures, and Rhat is the potential

scale reduction factor on split chains (at convergence, Rhat = 1).

Results were similar when we compared two intercept-only models, one with and one without the random effect of phylogeny. The estimated Bayes factor in favor of the non-phylogenetic model was 31.1, indicating strong evidence for a better fit of the non-phylogenetic model (Lee & Wagenmakers 2014).

The summary below shows the results of an intercept-only model with phylogeny fitted to the residual variance in the sex bias in return rate. The estimate of the standard deviation of the random effect of phylogeny was 0.13 with a wide 95% CI (0.01–0.40). The estimated Bayes factor in favor of the non-phylogenetic model was 17.4, again indicating clear evidence for a better fit of the non-phylogenetic model (Lee & Wagenmakers 2014).

Family: gaussian

Links: mu = identity; sigma = identity

Formula: Estimate ~ 0 + Intercept + (1 | species) + (1 | gr(phylogeny, cov = A))

Data: x (Number of observations: 64)

Samples: 5 chains, each with iter = 5000; warmup = 2500; thin = 1;

total post-warmup samples = 12500

Group-Level Effects:

~species (Number of levels: 32)

Estimate Est.Error l-95% CI u-95% CI Rhat Bulk_ESS Tail_ESS

sd(Intercept) 0.10 0.08 0.00 0.28 1.00 8156 5315

~phylogeny (Number of levels: 32)

Estimate Est.Error l-95% CI u-95% CI Rhat Bulk_ESS Tail_ESS

sd(Intercept) 0.13 0.11 0.01 0.40 1.00 6297 6505

Population-Level Effects:

Estimate Est.Error l-95% CI u-95% CI Rhat Bulk_ESS Tail_ESS

Intercept 0.03 0.13 -0.24 0.28 1.00 8641 7659

Family Specific Parameters:

Estimate Est.Error l-95% CI u-95% CI Rhat Bulk_ESS Tail_ESS

sigma 0.70 0.07 0.58 0.85 1.00 17408 9072

Samples were drawn using sampling(NUTS). For each parameter, Bulk_ESS

and Tail_ESS are effective sample size measures, and Rhat is the potential

scale reduction factor on split chains (at convergence, Rhat = 1).

Based on these results, we concluded that our models on return rate and sex bias in return rate did not need to incorporate the phylogeny.

**References**

- Bürkner, P-C. (2017). Brms: An *R* package for Bayesian multilevel models using *Stan*. *Journal of Statistical Software,* 80, 1–28. https://doi.org/10.18637/jss.v080.i01.
- Jetz, W., Thomas, G. H., Joy, J. B., Hartmann, K., & Mooers, A. O. (2012). The global diversity of birds in space and time. *Nature*, 491, 444–448.
- Lee M. D., & Wagenmakers E-J. (2014). Bayesian cognitive modeling: a practical course. Cambridge: Cambridge University Press.
- Paradis E., & Schliep K. (2019). ape 5.0: an environment for modern phylogenetics and evolutionary analyses in R. *Bioinformatics*, 35, 526–528.
- Revell, L. J. (2012). phytools: An R package for phylogenetic comparative biology (and other things). *Methods in Ecology and Evolution*, 3, 217–223. doi:10.1111/j.2041-210X.2011.00169.x
- Schliep K. P. (2011). phangorn: Phylogenetic analysis in R. *Bioinformatics*, 27, 592–593.

**C. Supplementary results**

**Table S1**. The mean posterior estimates of the beta coefficients for all predictors of the return rates of 49 species from 111 populations of shorebirds. Estimates are on a logit-scale and are back-transformed into ‘Probability’. The standard error and the 95% credible intervals are also included on the logit-scale. N = 175 observations.

|  | Estimate | Probability | Est.Error | l-95% | u-95% |
| --- | --- | --- | --- | --- | --- |
| Intercept | 0.060 | 0.515 | 0.243 | -0.419 | 0.541 |
| Male | 0.604 | 0.646 | 0.113 | 0.381 | 0.826 |
| Unknown | -0.437 | 0.393 | 0.190 | -0.814 | -0.067 |
| Polyandry | -2.025 | 0.117 | 0.814 | -3.659 | -0.438 |
| Polygyny | -1.942 | 0.125 | 0.629 | -3.191 | -0.732 |
| Wing length*_log_* | 0.451 | 0.611 | 0.178 | 0.102 | 0.798 |
| Number of birds banded*_log_* | -0.277 | 0.431 | 0.075 | -0.424 | -0.132 |
| Study year | -0.218 | 0.446 | 0.072 | -0.360 | -0.076 |
| Study duration | 0.052 | 0.513 | 0.112 | -0.168 | 0.271 |
| Study area size*_log_* | 0.019 | 0.505 | 0.086 | -0.151 | 0.188 |
| Relative latitude | -0.406 | 0.400 | 0.091 | -0.585 | -0.228 |
| Edge proximity | 0.015 | 0.504 | 0.083 | -0.148 | 0.176 |

**Table S2**. The mean posterior estimates of the beta coefficients for all predictors of the sex bias in return rates of 33 species from 65 populations of shorebirds. The first three models (1–3) considered the sex-bias in return rate as a continuous dependent variable with a beta distribution. The next three models (4–6) treated the sex-bias in return rate as a binary variable (0 if females returned more, 1 if males returned more). For each dependent variable, we ran a model with both mating system (categorical: monogamy, polyandry, polygyny) and sexual dimorphism in wing length (SSD) as fixed effects, and two models where each variable was included as a single fixed effect. In each model, posterior values of each observation were weighed by the total number of birds banded in that population, and species (or subspecies for species with multiple subspecies) was included as a random effect. The standard error and the 95% credible intervals are shown. N = 65 observations.

| Response | Model | Fixed | Variable | Estimate | Est.Error | l-95% | u-95% |
| --- | --- | --- | --- | --- | --- | --- | --- |
| Beta | 1 | Additive | Intercept | 0.359 | 0.143 | 0.077 | 0.642 |
|  |  |  | Polyandry | 0.634 | 0.514 | -0.370 | 1.645 |
|  |  |  | Polygyny | -0.109 | 0.525 | -1.155 | 0.918 |
|  |  |  | SSD | 0.044 | 0.129 | -0.211 | 0.301 |
|  | 2 | Mating system | Intercept | 0.363 | 0.141 | 0.089 | 0.646 |
|  |  |  | Polyandry | 0.562 | 0.464 | -0.337 | 1.489 |
|  |  |  | Polygyny | -0.025 | 0.469 | -0.953 | 0.885 |
|  | 3 | SSD | Intercept | 0.410 | 0.129 | 0.157 | 0.668 |
|  |  |  | SSD | -0.026 | 0.105 | -0.230 | 0.183 |
| Binary | 4 | Additive | Intercept | 6.303 | 2.464 | 3.163 | 12.355 |
|  |  |  | Polyandry | -0.671 | 3.709 | -7.539 | 7.078 |
|  |  |  | Polygyny | -5.119 | 7.926 | -22.206 | 9.254 |
|  |  |  | SSD | 2.649 | 2.013 | -0.276 | 7.457 |
|  | 5 | Mating system | Intercept | 5.462 | 1.852 | 2.897 | 9.997 |
|  |  |  | Polyandry | -3.912 | 2.657 | -9.500 | 1.148 |
|  |  |  | Polygyny | -0.772 | 4.365 | -8.079 | 9.645 |
|  | 6 | SSD | Intercept | 5.081 | 1.676 | 2.770 | 9.151 |
|  |  |  | SSD | 2.178 | 1.311 | 0.043 | 5.121 |

**Figure S1.** Scatterplot of pairwise correlations among predictor variables shown as Pearson’s correlation coefficients.


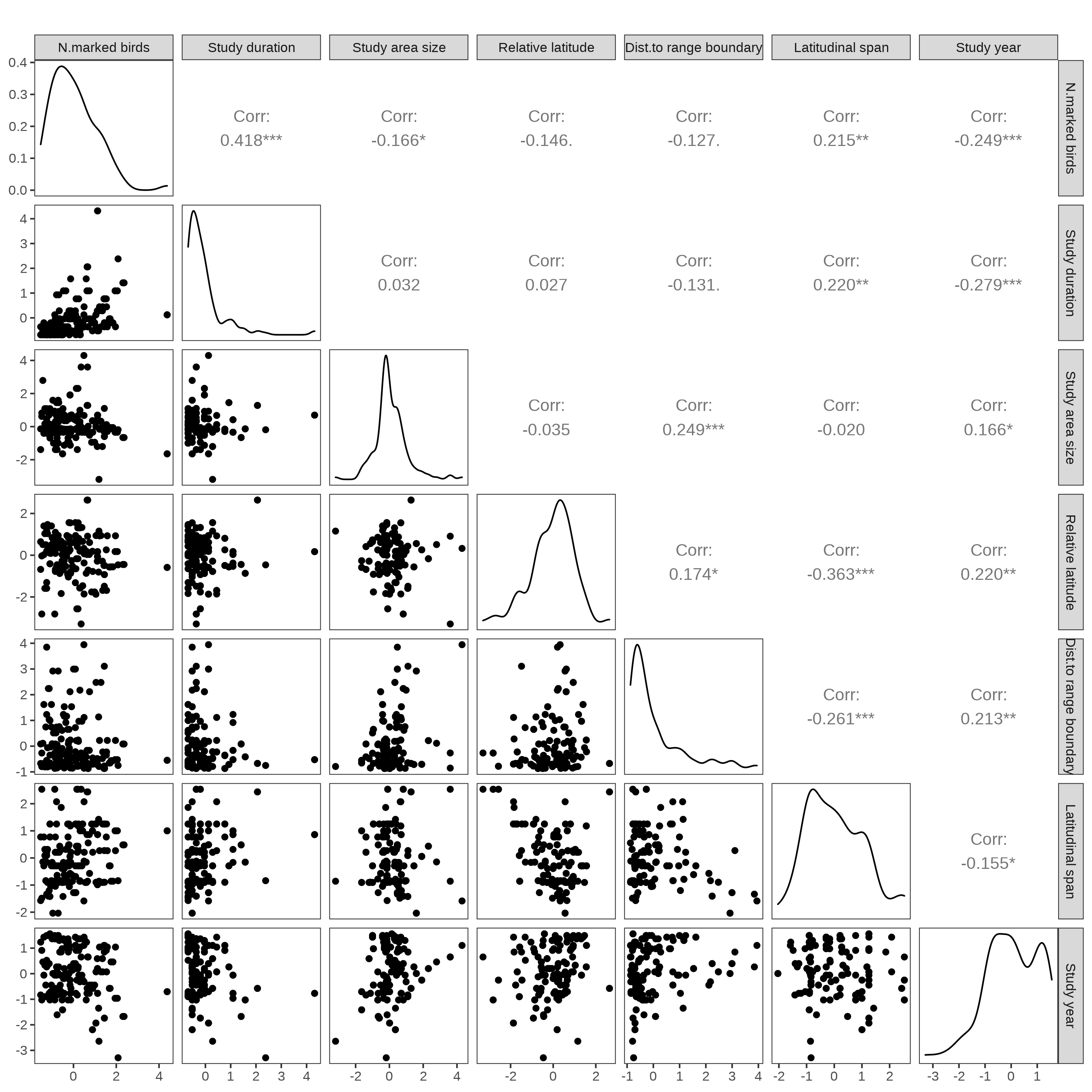


**Figure S2.** Trace plot of parameterized predictors from a Bayesian model fitted to the return rate of shorebirds. Model specification: 5 chains. 25000 warm-ups. 50000 iterations. with thinning rate of 5.


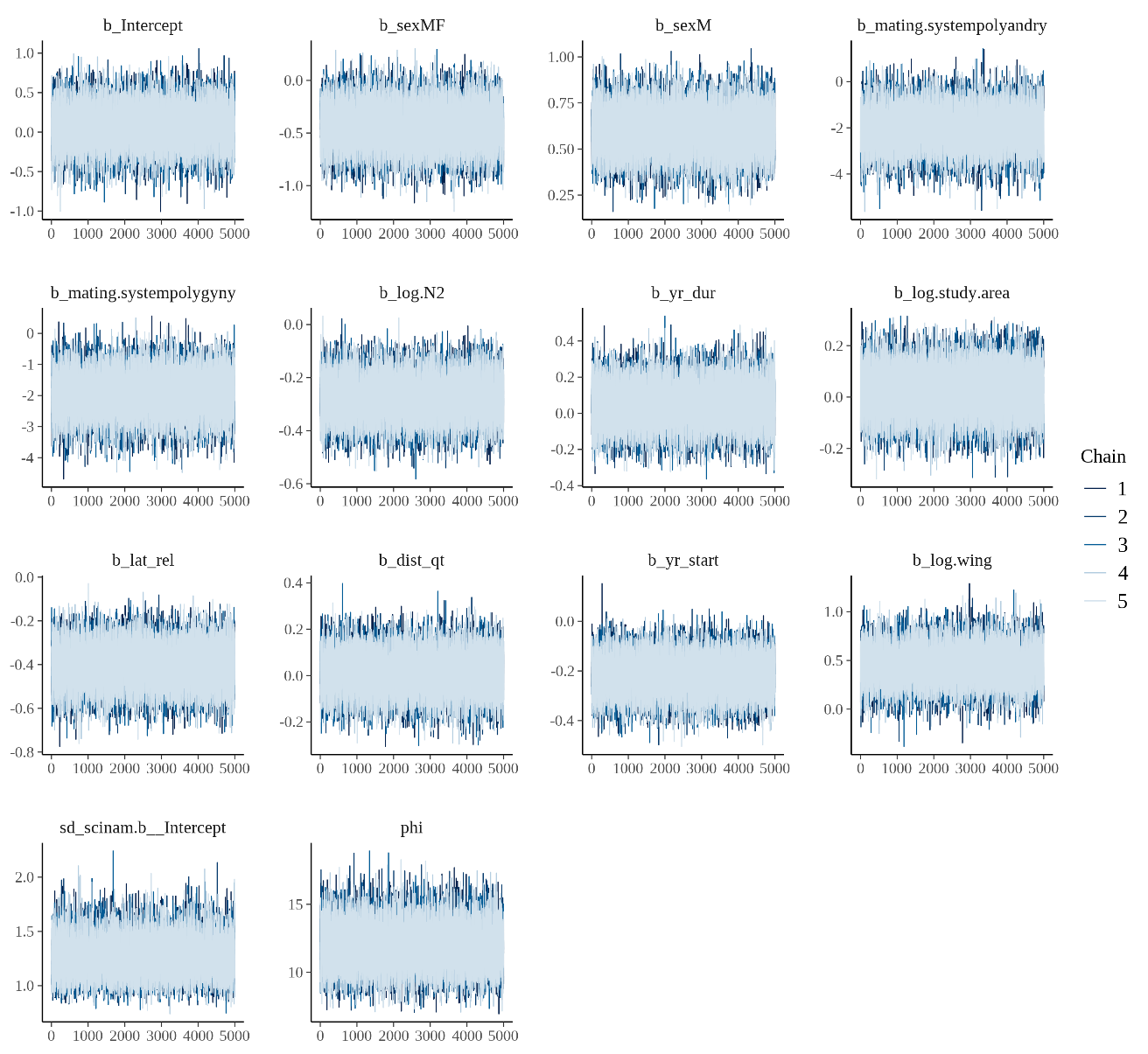


**Figure S3.** Trace plot of parameterized predictors from a Bayesian model with beta distribution and additive effects of the mating system and sexual size dimorphism fitted to the sex bias in return rate of shorebirds. Model specification: 5 chains. 25000 warm-ups. 50000 iterations. with thinning rate of 5.


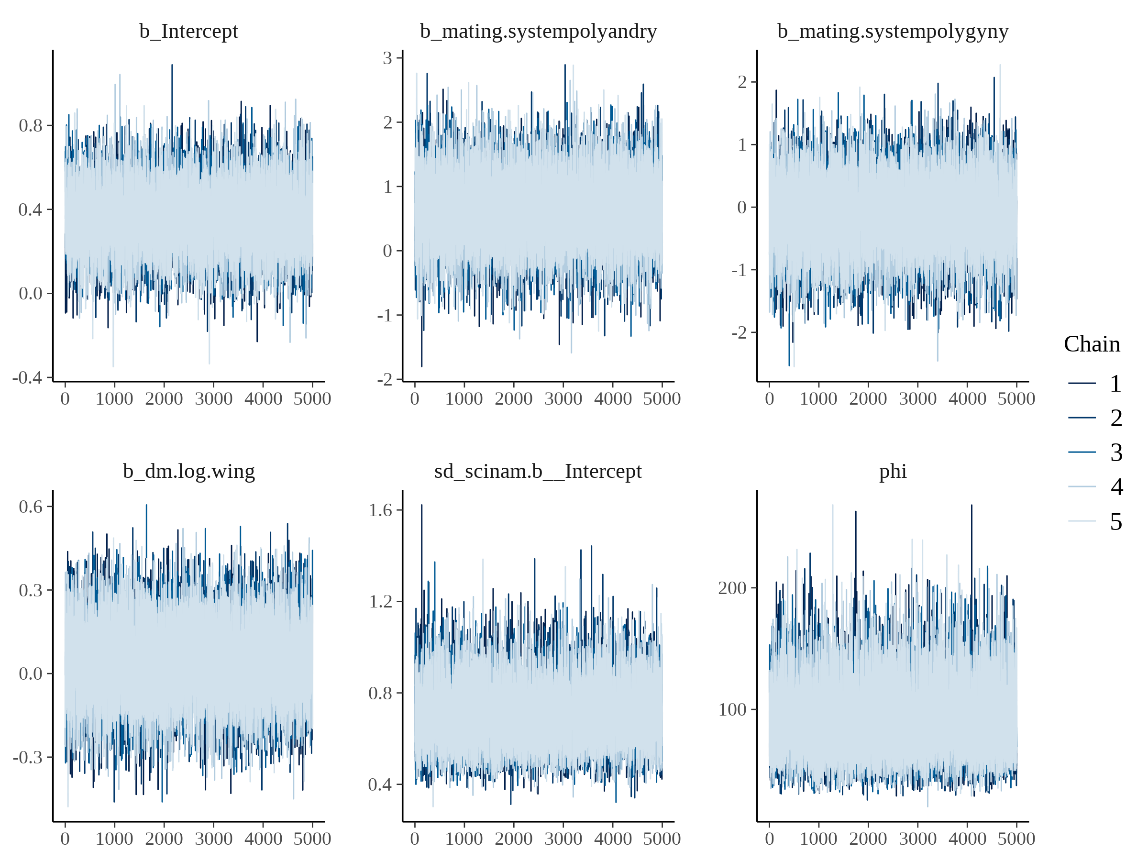


**Figure S4.** Comparison of standardized effect sizes (posterior means) of predictors explaining variation in annual return rates of shorebirds in relation to varying cut-offs of minimum sample size applied to the data. We fitted the same beta logistic regression model (see Methods in main text) to 17 different datasets, which included data points from populations of as little as 20 individuals up to at least 100 individuals. Error bars indicate 95% Bayesian credible intervals. A predictor has a significant effect if the 95% CI does not overlap zero. With higher cut-offs, the uncertainty of point estimates grew (shown by wider CI) due to the smaller sample size (number of included population estimates). However, the effect sizes themselves are robust to the changing cut-offs.


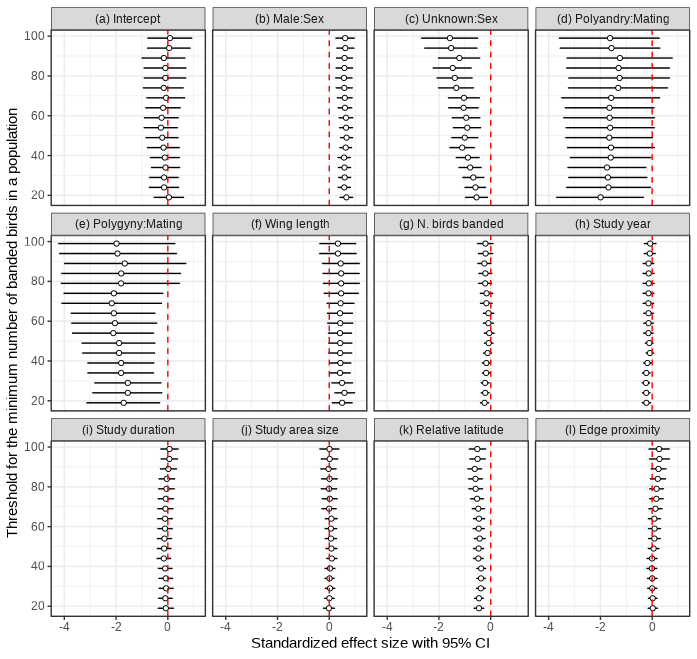


**D. Reference of data sources**

* Below references were used to extract the annual return rates of shorebirds. If interested in knowing the source of a particular data point, please, refer to our input data and the list of data sources archived in Dryad (doi:10.5061/dryad.1vhhmgqvd). Any source for a particular data point can be indexed with ‘sourceID’ column in the input data as well as the data source list.

Amat, J. A., Fraga, R. M., & Arroyo, G. M. (1999). Brood desertion and polygamous breeding in the Kentish Plover *Charadrius alexandrinus*. Ibis, 141, 596–607. https://doi.org/10.1111/j.1474-919X.1999.tb07367.x

Berg, Å. (1994). Maintenance of populations and causes of population changes of curlews *Numenius arquata* breeding on farmland. Biological Conservation, 67, 233–238. https://doi.org/10.1016/0006-3207(94)90614-9

Colwell, M. A., Burrell, N. S., Hardy, M. A., Kayano, K., Muir, J. J., Pearson, W. J., Peterson, S. A., & Sesser, K. A. (2010). Arrival times, laying dates, and reproductive success of Snowy Plovers in two habitats in coastal northern California. Journal of Field Ornithology, 81, 349–360.

Colwell, M. A., Gratto, C. L., Oring, L. W., & Fivizzani, A. J. (1988). Effects of blood sampling on shorebirds: Injuries, return rates, and clutch desertions. The Condor, 90, 942–945. https://doi.org/10.2307/1368855

Dougall, T. W., Holland, P. K., & Yalden, D. W. (2010). The population biology of Common Sandpipers in Britain. British Birds, 15.

Dwyer, T. J., Sepik, G. F., Derleth, E. L., & McAuley, D. G. (1988). Demographic Characteristics of a Maine Woodcock Population and Effects of Habitat Management. Fish and Wildlife Research 4. U.S. Fish and Wildlife Service, Patuxent Wildlife Research Center, Laurel, Maryland.

Eberhart-Phillips, L. J., Cruz-López, M., Lozano-Angulo, L., del Ángel, S. G., Rojas-Abreu, W., Bucio-Pacheco, M., & Küpper, C. (2020a). CeutaOPEN, individual-based field observations of breeding snowy plovers *Charadrius nivosus*. Scientific Data, 7, 149. https://doi.org/10.1038/s41597-020-0490-y

Eberhart-Phillips, L. J., Cruz-López, M., Lozano-Angulo, L., del Ángel, S. G., Rojas-Abreu, W., Bucio-Pacheco, M., & Küpper, C. (2020b). CeutaOPEN v1.5. Open Science Framework. DOI 10.17605/OSF.IO/3K4FHI.

Eichhorn, G., Bil, W., & Fox, J. W. (2017). Individuality in northern lapwing migration and its link to timing of breeding. Journal of Avian Biology, 48, 1132–1138. https://doi.org/10.1111/jav.01374

English, W., Norman, B., & Smith, P. (2020). Data on White-rumped Sandpiper contributed via personal communication.

Flynn, L., Nol, E., & Zharikov, Y. (1999). Philopatry, nest-site tenacity, and mate fidelity of Semipalmated Plovers. Journal of Avian Biology, 30, 47–55. https://doi.org/10.2307/3677242

Foppen, R. P. B., Majoor, F. A., Willems, F. J., Meininger, P. L., van Houwelingen, G. C., & Wolf, P. A. (2006). Survival and emigration rates in Kentish *Charadrius alexandrinus* and Ringed Plovers *Ch. Hiaticula* in the Delta area, SW-Netherlands. Ardea, 94, 159–173.

Grant, M. C. (1991). Nesting densities, productivity and survival of breeding Whimbrel *Numenius phaeopus* in Shetland. Bird Study, 38, 160–169. https://doi.org/10.1080/00063659109477085

Gratto-Trevor, C. (2020). Marbled Godwit (*Limosa fedoa*), version 1.0. In Birds of the World (A. F. Poole and F. B. Gill, Editors). Cornell Lab of Ornithology, Ithaca, NY, USA. <https://doi.org/10.2173/bow.margod.01>.

Gratto-Trevor, C. (2020). Data on Willets contributed via personal communication.

Groen, N. M., & Hemerik, L. (2002). Reproductive success and survival of Black-tailed Godwits *Limosa limosa* in a declining local population in The Netherlands. Ardea, 90, 239–248.

Haig, S. M., & Oring, L. W. (1988). Mate, site, and territory fidelity in Piping Plovers. The Auk, 105, 268–277. https://doi.org/10.2307/4087489

Handel, C. M., & Gill, R. E. (2000). Mate fidelity and breeding site tenacity in a monogamous sandpiper, the black turnstone. Animal Behaviour, 60, 471–481. https://doi.org/10.1006/anbe.2000.1505

Hazlitt, S. L., & Butler, R. W. (2001). Site fidelity and reproductive success of Black Oystercatchers in British Columbia. Waterbirds, 24, 203–207. https://doi.org/10.2307/1522031

Heldt, V. R. (1966). Zur Brutbiologie des Alpenstrandläufers, *Calidris alpina schinzii*. Corax, 1, 173–188.

Hildén, O., & Hilden, O. (1978). Population dynamics in Temminck’s Stint *Calidris temminckii*. Oikos, 30, 17–28. https://doi.org/10.2307/3543520

Hoefs, C. (2020). Data on Eurasian Dotterels contributed via personal communication.

Holland, P. K., & Yalden, D. W. (1991). Population dynamics of Common Sandpipers *Actitis hypoleucos* breeding along an upland river system. Bird Study, 38, 151–159. https://doi.org/10.1080/00063659109477084

Holmes, R. T. (1971). Density, habitat, and the mating system of the Western Sandpiper (*Calidris mauri*). Oecologia, 7, 191–208. https://doi.org/10.1007/BF00346361

Howe, M. A. (1982). Social Organization in a nesting population of Eastern Willets (*Catoptrophorus semipalmatus*). The Auk, 99, 88–102. https://doi.org/10.2307/4086024

Jackson, D. B. (1994). Breeding dispersal and site-fidelity in three monogamous wader species in the Western Isles, U.K. Ibis, 136, 463–473. https://doi.org/10.1111/j.1474-919X.1994.tb01123.x

Jehl, J. R. (1973). Breeding biology and systematic relationships of the Stilt Sandpiper. The Wilson Bulletin, 85, 34.

Jehl, J. R. (2007). Disappearance of breeding Semipalmated Sandpipers from Churchill, Manitoba: more than a local phenomenon. The Condor, 109, 351–360.

Johnson, J. (2020). Data on Red Knots contributed via personal communication.

Johnson, M., Ruthrauff, D. R., McCaffery, B. J., Haig, S. M., & Walters, J. R. (2010). Apparent survival of breeding Western Sandpipers on the Yukon-Kuskokwim river delta, Alaska. The Wilson Journal of Ornithology, 122, 15–22. https://doi.org/10.1676/09-089.1

Johnson, O. W., Bruner, P. L., Bruner, A. E., Johnson, P. M., Kienholz, R. J., & Brusseau, P. A. (2001). Features of breeding biology in Pacific and American golden-plovers nesting on the Seward Peninsula, Alaska. Wader Study Group Bulletin, 95, 59–65.

Jonsson, P. E. (1991). Reproduction and survival in a declining population of the Southern Dunlin *Calidris Alpina Schinzii*. Wader Study Group Bulletin, 61, 56–68.

Kålås, J. A., & Byrkjedal, I. (1984). Breeding chronology and mating system of the Eurasian Dotterel (*Charadrius morinellus*). The Auk, 101, 838–847. https://doi.org/10.2307/4086911

Klima, J., & Johnson, O. W. (2005). Return rates of male and female monogamous shorebirds to their breeding grounds. Wader Study Group Bulletin, 106, 42–46.

Kosztolányi, A., Javed, S., Küpper, C., Cuthill, I. C., Shamsi, A. A., & Székely, T. (2009). Breeding ecology of Kentish Plover *Charadrius alexandrinus* in an extremely hot environment. Bird Study, 56, 244–252. https://doi.org/10.1080/00063650902792106

Lanctot, R. B., Brown, S. C., & Sandercock, B. K. (2016). Arctic Shorebird Demographics Network. NSF Arctic Data Center. doi:10.18739/A2222R68W

Lanctot, R. B., Scribner, K. T., Lanctot, R. B., Weatherhead, P. J., & Kempenaers, B. (1997). Lekking without a paradox in the Buff‐breasted Sandpiper. The American Naturalist, 149, 1051–1070. https://doi.org/10.1086/286038

Lanctot, R. B., Weatherhead, P. J., Kempenaers, B., & Scribner, K. T. (1998). Male traits, mating tactics and reproductive success in the Buff-breasted Sandpiper, *Tryngites subruficollis*. Animal Behaviour, 56, 419–432. https://doi.org/10.1006/anbe.1998.0841

Lishman, C., Nol, E., Abraham, K. F., & Nguyen, L. P. (2010). Behavioral responses to higher predation risk in a subarctic population of the Semipalmated Plover. The Condor, 112, 499–506. https://doi.org/10.1525/cond.2010.090059

Lislev, T., Byrkjedal, I., & Grønstøl, G. B. (2008). Dispersal and age at first breeding in Norwegian Northern Lapwings (*Vanellus vanellus*). Ornis Fennica, 86, 11–17.

Lislevand, T., & Hahn, S. (2013). Effects of geolocator deployment by using flexible leg-loop harnesses in a small wader. Wader Study Group Bulletin, 120, 108–113.

Loegering, J. P. (1992). Piping plover breeding biology, foraging ecology and behavior on Assateague Island National Seashore, Maryland. M.S. thesis, Virginia Polytechnic and State University, Blacksburg, VA.

Lowther, P. E., Douglas, H. D., & Gratto-Trevor, C. (2020). Willet (*Tringa semipalmata*), version 1.0. In Birds of the World (A. F. Poole and F. B. Gill, Editors). Cornell Lab of Ornithology, Ithaca, NY, USA. https://doi.org/10.2173/bow.willet1.01.

Marks, J. S., Tibbitts, T. L., Gill, R. E., & McCaffery, B. J. (2020). Bristle-thighed Curlew (*Numenius tahitiensis*). Birds of the World. https://birdsoftheworld.org/bow/species/brtcur/cur/introduction

Mayer, P. M., & Ryan, M. R. (1986). Population ecology of the Piping Plover in the northern Great Plains. U.S. Fish and Wildlife Service. https://www.fws.gov/northeast/pipingplover/pdf/I.pdf

Miller, E. H. (1983). Habitat and breeding cycle of the Least Sandpiper (*Calidris minutilla*) on Sable Island, Nova Scotia. Canadian Journal of Zoology, 61, 2880–2898. https://doi.org/10.1139/z83-376

Moitoret. (1996). Predevelopment surveys of nesting birds at two sites in the Kuparuk oilfield, Alaska, 1988–1992. Fish and Wildlife Service Technical Report NAES-TR-96-02.

Mong, T. W., & Sandercock, B. K. (2007). Optimizing radio retention and minimizing radio impacts in a field study of Upland Sandpipers. Journal of Wildlife Management, 71, 971–980. https://doi.org/10.2193/2005-775

Moskoff, W., & Montgomerie, R. (2020). Baird’s Sandpiper (*Calidris bairdii*), version 1.0. In Birds of the World (A. F. Poole and F. B. Gill, Editors). Cornell Lab of Ornithology, Ithaca, NY, USA. https://doi.org/10.2173/bow.baisan.01.

Nol, E. (1985). Sex roles in the American Oystercatcher. Behaviour, 95, 232–260. https://doi.org/10.1163/156853985X00145

Oring, L. W., & Lank, D. B. (1982). Sexual selection, arrival times, philopatry and site fidelity in the polyandrous spotted sandpiper. Behavioral Ecology and Sociobiology, 10, 185–191. https://doi.org/10.1007/BF00299684

Oring, L. W., Lank, D. B., & Maxson, S. J. (1983). Population studies of the polyandrous Spotted Sandpiper. The Auk, 100, 272–285.

Page, G. W., Stenzel, L. E., Winkler, D. W., & Swarth, C. W. (1983). Spacing out at Mono Lake: Breeding success, nest density, and predation in the Snowy Plover. The Auk, 100, 13–24. https://doi.org/10.1093/auk/100.1.13

Pakanen, V.-M., Hildén, O., Rönkä, A., Belda, E. J., Luukkonen, A., Kvist, L., & Koivula, K. (2011). Breeding dispersal strategies following reproductive failure explain low apparent survival of immigrant Temminck’s stints. Oikos, 120, 615–622. https://doi.org/10.1111/j.1600-0706.2010.18953.x

Pakanen, V.-M., Lampila, S., Arppe, H., & Valkama, J. (2015). Estimating sex specific apparent survival and dispersal of Little Ringed Plovers (*Charadrius dubius*). Ornis Fennica, 92, 172–186.

Pakanen, V.-M., Rönkä, N., Thomson, R. L., & Koivula, K. (2015). No strong effects of leg-flagged geolocators on return rates or reproduction of a small long-distance migratory shorebird. Ornis Fennica, 92, 101–111.

Parr, R. (1992). The decline to extinction of a population of Golden Plover in North-East Scotland. Ornis Scandinavica (Scandinavian Journal of Ornithology), 23, 152–158. https://doi.org/10.2307/3676443

Paton, P., & Edwards, T. (1996). Factors affecting interannual movements of Snowy Plovers. The Auk, 113, 534–543. https://doi.org/10.2307/4088973

Pearce-Higgins, J. W., & Yalden, D. W. (2003). Golden Plover *Pluvialis apricaria* breeding success on a moor managed for shooting Red Grouse *Lagopus lagopus*. Bird Study, 50, 170–177. https://doi.org/10.1080/00063650309461309

Perkins, D. E., Smith, P. A., & Gilchrist, H. G. (2007). The breeding ecology of ruddy turnstones (*Arenaria interpres*) in the eastern Canadian Arctic. Polar Record, 43, 135–142. https://doi.org/10.1017/S0032247407006092

Pienkowski, M. W. (1984). Behaviour of young Ringed Plovers *Charadrius hiaticula* and its relationship to growth and survival to reproductive age. Ibis, 126, 133–155. https://doi.org/10.1111/j.1474-919X.1984.tb07994.x

Ray, K. L. (2010). Factors affecting Wilson’s Plover (*Charadrius wilsonia*) demography and habitat use at Onslow Beach, Marine Corps Base Camp Lejeune, North Carolina. M.S. thesis, Virginia Polytechnic and State University, Blacksburg, VA.

Redmond, R. L., & Jenni, D. A. (1982). Natal philopatry and breeding area fidelity of Long-billed Curlews (*Numenius americanus*): Patterns and evolutionary consequences. Behavioral Ecology and Sociobiology, 10, 277–279. https://doi.org/10.1007/BF00302817

Reed, J. M., & Oring, L. W. (1993). Philopatry, site fidelity, dispersal, and survival of Spotted Sandpipers. The Auk, 110, 541–551. https://doi.org/10.2307/4088418

Reynolds, J. D., & Cooke, F. (1988). The influence of mating systems on philopatry: A test with polyandrous Red-necked Phalaropes. Animal Behaviour, 36, 1788–1795. https://doi.org/10.1016/S0003-3472(88)80118-0

Rittinghaus, H. (1956). Untersuchungen am Seeregenpfeifer (*Charadrius alexandrinus* *L*.) auf der Insel Oldeoog. Journal für Ornithologie, 97, 117–155. https://doi.org/10.1007/BF01671028

Robinson, J. A., & Oring, L. W. (1997). Natal and breeding dispersal in American Avocets. The Auk, 114, 416–430. https://doi.org/10.2307/4089243

Roche, E. A., Sherfy, M. H., Ring, M. M., Shaffer, T. L., Anteau, M. J., & Stucker, J. H. (2016). Demographics and movements of Least Terns and Piping Plovers in the Central Platte River Valley, Nebraska: U.S. Geological Survey Open-File Report 2016–1061, 27 p., http://dx.doi.org/10.3133/ofr20161061.

Rodrigues, T. M. G. (2020). Data on Common Snipes contributed via personal communication.

Ryabitsev, V. K. (2000). On the population ecology of the Gray Plover in the northern Yamal Peninsula. Russian Journal of Ecology, 31, 108–112. https://doi.org/10.1007/BF02828364

Safriel, U. N., Harris, M. P., Brooke, M. D. L., & Britton, C. K. (1984). Survival of breeding Oystercatchers *Haematopus ostralegus*. Journal of Animal Ecology, 53, 867–877. https://doi.org/10.2307/4664

Sandercock, B. K., & Gratto‐Trevor, C. L. (1997). Local survival in Semipalmated Sandpipers *Calidris pusilla* breeding at La Pérouse Bay, Canada. Ibis, 139, 305–312. https://doi.org/10.1111/j.1474-919X.1997.tb04629.x

Sandercock, B. K., Székely, T., & Kosztolányi, A. (2005). The effects of age and sex on the apparent survival of Kentish Plovers breeding in Southern Turkey. The Condor, 107, 583–596.

Schamel, D., & Tracy, D. M. (1991). Breeding site fidelity and natal philopatry in the sex role-reversed Red and Red-Necked Phalaropes (Fidelidad al Área de Reproducción y Filopatría Natal en Phalaropus fulicaria y P. lobatus). Journal of Field Ornithology, 62, 390–398.

Senner, N. R., Hochachka, W. M., Fox, J. W., & Afanasyev, V. (2014). An exception to the rule: Carry-over effects do not accumulate in a long-distance migratory bird. PLOS ONE, 9(2), e86588. https://doi.org/10.1371/journal.pone.0086588

Senner, N. R., & Swift, R. (2020). Data on Hudsonian Godwits contributed via personal communication.

Sheldon, R. D., Kamp, J., Koshkin, M. A., Urazaliev, R. S., Iskakov, T. K., Field, R. H., Salemgareev, A. R., Khrokov, V. V., Zhuly, V. A., Sklyarenko, S. L., & Donald, P. F. (2013). Breeding ecology of the globally threatened Sociable Lapwing *Vanellus gregarius* and the demographic drivers of recent declines. Journal of Ornithology, 154, 501–516. https://doi.org/10.1007/s10336-012-0921-4

Skeel, M. A. (1983). Nesting success, density, philopatry, and nest-site selection of the Whimbrel (*Numenius phaeopus*) in different habitats. Canadian Journal of Zoology, 61, 218–225. https://doi.org/10.1139/z83-027

Sviridova, T. (2000). Site fidelity and territory establishment in breeding Pacific Golden Plovers and Grey Plovers at Taimyr, Siberia. Heritage of the Russian Arctic: Research, Conservation, and International Co-operation. Moscow: Ecopros Publishers.

Székely, T., & Williams, T. D. (1995). Costs and benefits of brood desertion in female Kentish Plovers, *Charadrius alexandrinus*. Behavioral Ecology and Sociobiology, 37, 155–161. https://doi.org/10.1007/BF00176712

Thompson, P. S., & Hale, W. G. (1993). Adult survival and numbers in a coastal breeding population of Redshank *Tringa totanus* in northwest England. Ibis, 135, 61–69. https://doi.org/10.1111/j.1474-919X.1993.tb02810.x

Thorup, O. (1999). Breeding dispersal and site-fidelity in Dunlin *Calidris alpina* at Tipperne, Denmark. Journal of the Danish Ornithological Society, 93, 255–265.

Thuman, K. (2003). Female Reproductive Strategies in the Ruff (*Philomachus pugnax*). PhD dissertation. University of Uppsala, Uppsala, Sweden.

Tomkovich, P. S., & Soloviev, M. Y. (1994). Site fidelity in high Arctic breeding waders. Ostrich, 65, 174–180. https://doi.org/10.1080/00306525.1994.9639680

Tomkovich, P.S. (1996). A third report on the biology of the Great Knot, *Calidris tenuirostris*, on the breeding grounds. Stilt, 28, 43.

van Leeuwen, C. H. A., & Jamieson, S. E. (2018). Strong pair bonds and high site fidelity in a subarctic-breeding migratory shorebird. The Wilson Journal of Ornithology, 130, 140–151. https://doi.org/10.1676/16-116.1

Warriner, J. S., Warriner, J. C., Page, G. W., & Stenzel, L. E. (1986). Mating system and reproductive success of a small population of polygamous Snowy Plovers. Wilson Bulletin, 98, 15–37.

Weiser, E. L., Lanctot, R. B., Brown, S. C., Gates, H. R., Bentzen, R. L., Bety, J., Boldenow, M. L., English, W. B., Franks, S. E., Koloski, L., Kwon, E., Lamarre, J.-F., Lank, D. B., Liebezeit, J. R., McKinnon, L., Nol, E., Rausch, J., Saalfeld, S. T., Senner, N. R., … Sandercock, B. K. (2018). Environmental and ecological conditions at Arctic breeding sites have limited effects on true survival rates of adult shorebirds. Auk, 135, 29–43.

Wiens, T. P. (1986). Nest-site tenacity and mate retention in the Piping Plover. MS Thesis, University of Minnesota at Duluth. Duluth, MN 55812, United States.

Wilcox, L. R. (1959). A twenty year banding study of the Piping Plover. The Auk, 76, 129–152. https://doi.org/10.2307/4081772

Yasué, M., & Dearden, P. (2008). Replacement nesting and double-brooding in Malaysian Plovers *Charadrius peronii*: Effects of season and food availability. Ardea, 96, 59–72. https://doi.org/10.5253/078.096.0107

Ye, Z. M. (1992). Territorial relations and population structure of the Redshank *Tringa totanus* during the nesting period in the south of Ukraine. Wader Study Group, 64, 45–46.

Yezerinac, S., Lanctot, R. B., Sage, G. K., & Talbot, S. L. (2013). Social and genetic mating system of the American Golden-Plover. Condor, 115, 808–815.

Zöckler, C., Syroechkovskiy, E. E., & Atkinson, P. W. (2010). Rapid and continued population decline in the Spoon-billed Sandpiper *Eurynorhynchus pygmeus* indicates imminent extinction unless conservation action is taken. Bird Conservation International, 20, 95–111. https://doi.org/10.1017/S0959270910000316
